# Effect of Polydextrose on the Cooking and Gelatinization Properties and Microstructure of Chinese Early Indica Rice

**DOI:** 10.3390/gels11030171

**Published:** 2025-02-26

**Authors:** Mengya Wang, Chang Liu, Xiaohong Luo, Jianzhang Wu, Xingjun Li

**Affiliations:** 1 College of Grain and Strategic Reserves, Henan University of Technology, Zhengzhou 450001, China; ahueqw1007@163.com (M.W.); qlsszz@126.com (J.W.); 2 Academy of National Food and Strategic Reserves Administration, National Engineering Research Center for Grain Storage and Transportation, Beijing 102209, China; shlglc@126.com (C.L.); lxh@ags.ac.cn (X.L.); 3 College of Health Science and Engineering, University of Shanghai for Science and Technology, Shanghai 200093, China

**Keywords:** early indica rice, polydextrose, cooking test, cooked rice retrogradation, Mixolab test, microstructure

## Abstract

To reduce the hard texture of cooked early indica rice, two types of polydextrose (ST with 1% moisture content (MC) and XG with 4.7% MC) were added at 0%, 3%, 5%, 7%, and 10%, respectively, to the cooking milled rice polished from the paddies of the 2.5-year-stored IP46 variety and the newly harvested Sharuan Nian (SRN) variety. Compared with early indica rice without polydextrose, the cooking time was significantly reduced and gruel solids loss was increased with the increase in polydextrose addition. Generalized linear model (GLM) analysis shows that both polydextrose equally reduced the hardness, adhesive force, adhesiveness, cohesiveness, gumminess, and chewiness of the cooked early indica rice, and maintained the resilience. They also significantly reduced the rapid viscosity analysis (RVA) parameters like the peak viscosity, trough viscosity, breakdown viscosity, final viscosity, and setback viscosity of early indica rice, and significantly increased the peak time and pasting temperature. Both polydextrose significantly increased the gelatinization temperature of rice flour measured by a differential scanning calorimeter (DSC)and reduced the gelatinization enthalpy and aging. Compared with the sample without polydextrose, the addition of two types of polydextrose significantly increased the dough development time of rice flour measured by a Mixolab, but reduced the maximum gelatinization torque, starch breakdown and setback torque, and heating rate. XG had a higher capability in decreasing the rice cooking time and the aging of retrograded rice flour paste, and in increasing the score of the appearance structure and taste in cooked rice than ST; ST was better in decreasing the gelatinization enthalpy of rice flour paste and the setback torque of rice dough than XG, maybe due to the polymer molecular weight. Microstructure analysis showed that adding polydextrose promoted the entry of water molecules into the surface of the rice kernel and the dissolution of starch, and the honeycomb structure was gradually destroyed, resulting in larger pores. The cross-section of the cooked rice kernel formed cracks due to the entry of water, the cracks in the IP46 variety were larger and shallower than those in the SRN variety, and there were more filamentous aggregates in the IP46 variety. Polydextrose addition aggravated the swelling of starch granules, made the internal structure loose and produced an obvious depression in the central area of the cross-section, forming soft and evenly swollen rice kernels. These results suggest that polydextrose addition can significantly improve the hard texture of cooked early indica rice and shorten the cooking time.

## 1. Introduction

Rice is one of the world’s major food staples and is recognized as the most important food crop in Asia. To meet the food demand of the world’s growing population, rice production needs to be continuously increased [1,2]. In China, the rice planting system is usually single-season or double-season rice (early rice and late rice), while in other southeast Asian countries, it is wet-season and dry-season rice [3,4]. The area of double-season rice planting accounts for 33% of the rice planting area in China, and its yield accounts for 27% of the total rice yield [5]. China produces about28 million tons of early indica rice every year, part of which enters the storage link for two to three years’ storage due to its large population, and the rest is used as food, processing materials, or feed. Early indica rice has a harder texture when cooked, and there are fewer studies on the modification of cooked early indica rice.

Early indica rice has a dietary fiber content of 0.6–1.3% [6], and rice is a typical low-dietary-fiber and high-glycemic-index staple food [7]. Studies have shown that the consumption of rice as a staple food is significantly associated with the high incidence of type 2 diabetes in east and southeast Asia regions [8]. At present, adding dietary fiber to food is the main means to supplement the insufficient daily intake of dietary fiber in a human body. The hydrolysis of starch in wheat flour with added dietary fiber is significantly reduced, and this food is a low-glycemic-index food that is beneficial to human health [9]. However, for rice, the common way people eat it is to cook the rice grains and then eat them, and it is worth studying the addition of exogenous dietary fiber in cooking rice.

Dietary fiber is one of the significant components that may reduce the glycemic index of food through lowering postprandial blood glucose [10]. Polydextrose is a recognized water-soluble dietary fiber in more than 20 countries and beneficial to human health [11,12]. Compared with insoluble dietary fiber, polydextrose has more health functions and processing advantages. Polydextrose can be widely used in various foods, especially in low-energy, high-fiber functional foods, because it has low calories, stability, and extremely high tolerance [12]. It belongs to hydrocolloids that can provide viscosity in the food industry [13]. Polydextrose can be used as a humectant in food, for example, in baked food, polydextrose can delay its evaporation, prevent the product from going off, and keep or extend the shelf life of the product [14]. There is a lack of research on the addition of polydextrose in cooking early indica rice and its effect on the aging of rice kernels.

The interaction of polysaccharides to pure rice starch has been paid more attention in recent years [15,16]. Chen et al. [17] suggested that pullulan can not only inhibit the short-term retrogradation of amylose in rice starch by inhibiting the swelling and gelatinization of rice starch but also inhibit the long-term retrogradation of its amylopectin by slowing down the movement and association between starch molecular chains. Based on an assumption that the addition of polydextrose, an amorphous powder with strong hygroscopicity and weakly acidic, water-soluble nature [13], could promote the gelatinization of starch during the cooking of early indica rice, the present study investigated the cooking, textural, pasting, thermal, and thermo-mechanical properties of two indica rice samples with the addition of1% or 4.7% MC polydextrose, one rice sample milled from an indica variety of paddy stored for 2.5 years, and the other sample from a newly harvested indica paddy variety, and their cooked rice microstructure, with the aim to improve the taste texture and shelf life of the cooked early indica rice rich in dietary fiber and thus maintain human health.

## 2. Results and Discussion

### 2.1. Effect of Adding Polydextrose on the Cooking Characteristics of Early Indica Rice

**Figure 1:**
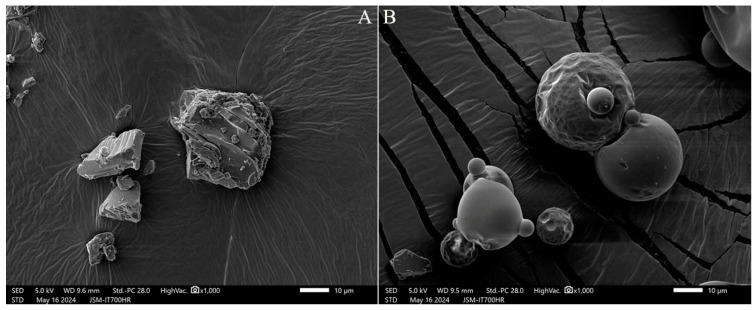
Scanning electron micrographs of two types of polydextrose. Note: (**A**) ST polydextrose (1.0% moisture content, MC); (**B**) XG polydextrose (4.7% MC). All photos are enlarged 1000×.

The present study used two types of polydextrose with different synthetic processes; ST types are uneven irregular particles and XG types are uneven spherical particles (Figure 1), and their moisture content was 1.0% and 4.7%, respectively. The polydextrose with a lower moisture content has a high cost performance in the market.

Table 1 shows the effect of adding polydextrose (PD) on the cooking properties of two early indica rice. The optimal cooking time and gruel solids loss of the IP46 rice milled from the 2.5-year-stored paddy in a warehouse were significantly (*p* < 0.05) lower than those of the SRN rice from the newly harvested indica paddy. With the increase in the additional content of polydextrose, the cooking time of the rice and gruel solids loss were significantly reduced and increased, respectively. With regard to the modification effect of polydextrose types, XG was stronger than ST. In terms of early indica rice varieties, the response of the SNR variety to the modification of polydextrose was stronger than that of IP46. The water uptake ratio of the rice did not change significantly with the increase in polydextrose addition. The cooked rice with the addition of10% polydextrose had the same color as the control sample without polydextrose.

A GLM analysis (Table 2) confirms that the SRN rice had a significantly (*p* < 0.05) shorter cooking time than IP46, and showed a greater gruel solids loss than IP46. Compared with ST, XG significantly reduced the cooking time and water uptake ratio of the two early indica rice. A 3–10% polydextrose addition significantly reduced their cooking time and increased the gruel solids loss.

### 2.2. Effect of Adding Polydextrose on the Texture of Cooked Early Indica Rice

Table 3 shows the effect of adding polydextrose on the texture of the two cooked early indica rice. With the increase in polydextrose addition, the hardness of the cooked IP46 rice was kept by 3–7% ST and 3%, 7–10% XG except for 10% ST, and 5% XG, while that of the SRN cooked rice decreased. Compared with the SRN rice, the longer cooking time and the more difficult polydextrose-modified cooking hardness in the IP 46 rice might be due to its biochemical components and 2.5-year storage time. The adhesive force, adhesiveness, cohesiveness, springiness, gumminess, and chewiness of the cooked IP46 rice remained unchanged with the increase in polydextrose addition, while these parameters in the cooked SRN rice decreased. The resilience in both cooked rice remained unchanged with the increase in polydextrose addition.

A GLM analysis (Table 4) confirms that the hardness, adhesive force, adhesiveness, springiness, gumminess, and chewiness of the cooked SRN rice were significantly (*p* < 0.05)greater than those of the cooked IP46 rice, but the resilience and cohesiveness were less than those of the cooked P46 rice. Compared with the cooked rice sample without polydextrose, two types of polydextrose addition equally reduced the hardness, adhesive force, adhesiveness, cohesiveness, gumminess, and chewiness of the cooked early indica rice. ST maintained the springiness of the cooked rice, while XG significantly reduced it; a 5–10% polydextrose addition maintained the springiness of the cooked early indica rice. Two types of polydextrose addition equally maintained the resilience of the cooked early indica rice. Compared with the addition of 0% polydextrose, a 5% addition significantly reduced the resilience of the cooked early indica rice.

### 2.3. Effect of Adding Polydextrose on the Pasting Property of Early Indica Rice Flours

Table 5 shows the effect of adding polydextrose on the pasting property of two early indica rice flours. With the increase in polydextrose addition, the peak viscosity, trough viscosity, final viscosity, breakdown viscosity, and setback viscosity in the pasting characteristics of two early indica rice varieties were significantly (*p* < 0.05) reduced, while the peak time and pasting temperature increased. The effect of the XG addition was greater than that of the ST addition. The decrease in the setback viscosity indicates that adding polydextrose has an inhibitory effect on the starch retrogradation of rice flours.

A GLM analysis (Table 6) confirms that the peak viscosity and breakdown viscosity of the SRN rice paste were greater than those of the IP46 rice paste, while the trough viscosity, final viscosity, setback viscosity, peak time, and pasting temperature were significantly (*p* < 0.05) lower than those of the IP46 rice paste. Both types of polydextrose addition significantly reduced the peak viscosity, trough viscosity, breakdown viscosity, final viscosity, and setback viscosity of the early indica rice flours, and significantly increased their peak time and pasting temperature. This might be due to the possibility that the binding force between polydextrose and water molecules is greater than that between starch and water molecules in rice flour paste, which weakens the hydrogen bond interaction between starch and water molecules, enhances the interaction between starch molecules, and leads to the decrease in the paste viscosity of rice flour and the increase in the pasting temperature, indicating that the pasting process becomes slow.

The breakdown viscosity of the rice flour paste/polydextrose system was significantly reduced, which may indicate that polydextrose reduced the leakage of amylose from starch granules and improved the stability of the rice flour paste, but our cooking test showed that the gruel solids loss increased, meaning that the addition of polydextrose did not prevent the leakage of amylose in the cooked early indica rice. The setback viscosity of this system was significantly reduced, indicating that the ability of the molecules, especially amylose, in rice flour paste after pasting, to re-order through hydrogen bonds during the cooling process was reduced, and adding polydextrose played a role in the short-term aging of the rice flour paste.

### 2.4. Effect of Adding Polydextrose on the Thermal Property of Early Indica Rice Flours

To further determine the influence of adding polydextrose on the thermal properties of two early indica rice, the DSC gelatinization parameters of the hydrated rice flour sample coated with a crucible were measured at day 0 (Table 7) and day 21 (Table 8), respectively. At day 0, the increase in polydextrose addition did not significantly affect the gelatinization enthalpy of the two early indica rice flours, but significantly (*p* < 0.05) increased the peak temperature. The onset temperature increased with the increase in polydextrose addition, which was not significant for the IP46 rice, but was significant for the SRN rice. The conclusion temperature of the IP46 rice did not decrease significantly with the increase in polydextrose addition, while that of the SRN rice decreased significantly. The width of the gelatinization peak showed a similar trend to the *T*_c_ with the increase in polydextrose addition. The height of the gelatinization peak decreased with the increase in polydextrose addition.

At the 21-day measurement, the gelatinization enthalpy and peak width and height decreased significantly (*p* < 0.05) with the polydextrose addition, but the *T*_p_, *T*_o,_ and *T*_c_ increased with the polydextrose addition (Table 8). The aging rate of the rice four paste was expressed as the enthalpy ratio percentage and *T*_c_ reduction percent, and the aging rate decreased with the increase in polydextrose addition, and XG had a better inhibition effect on the aging rate of the early indica rice flour paste than ST. The aging rate of the rice flour paste expressed by the enthalpy ratio percentage was better than that expressed by the reduction percent of *T*_c_ (Table 9).

A GLM analysis of the thermal properties in the early indica rice flours at day 0 (Table 10) confirms that the *T*_p_, *T*_o_, *T*_c_, and peak width of the gelatinization of the IP46 rice were all greater than those of the SRN rice, but the peak height was less than that of the SRN rice, and their gelatinization enthalpies were similar. Compared with the sample without polydextrose, two types of polydextrose addition significantly (*p* < 0.05) increased the *T*_p_ of rice flours and significantly decreased the *T*_c_ and the width of the gelatinization peak. XG maintained the gelatinization enthalpy, *T*_o_, and gelatinization peak height of the rice flours, while ST significantly these three parameters. A 5–10% polydextrose addition significantly reduced the gelatinization enthalpy but increased the *T*_o_; a 3–10% polydextrose addition significantly reduced the gelatinization peak height. Due to the lower moisture content of ST polydextrose compared to XG, ST might more strongly inhibit the starch molecules in rice flours from uptaking water, swelling, and gelatinizing during the competition for water molecules. The configuration of the starch crystalline and amorphous regions was changed, resulting in a lower enthalpy required for fusion.

A GLM analysis of the thermal properties in the early indica rice flours at day 21 (Table 11) further shows that the 3–10% polydextrose addition reduced the gelatinization enthalpy and the peak height and width, but increased the onset temperature, peak temperature, and conclusion temperature of gelatinization. The aging rate of IP46 was greater than that of SRN; compared with the sample without polydextrose, ST addition maintained the aging rate, while XG reduced the aging rate, and a 5–10% polydextrose addition significantly reduced the aging rate of the early indica rice paste. This is mainly due to the small molecular weight of polydextrose and the presence of many hydroxyl groups in its molecular structure, which could combine with the short side chains of some branched starch molecules in the rice flour paste during refrigeration, hindering the recrystallization of branched starch to form an orderly crystal structure, and the heat required for crystal melting is reduced; thus, the degree of starch gel retrogradation in early indica rice is reduced.

### 2.5. Effect of Polydextrose on Thermo-Mechanical Property of Early Indica Rice Dough

Table 12 shows the effect of adding polydextrose on the thermo-mechanical property of flour dough in the early indica rice. The addition of 3–7% polydextrose reduced the development time (DDT) and stable time (DST) of the IP46 rice flour dough but maintained or even increased the DDT and DST of the SRN rice flour dough. Polydextrose addition increased the protein weakness degree (C1–Cs) of the IP46 rice flour dough but reduced the protein weakening degree of the SRN rice flour dough. The addition of polydextrose significantly reduced the maximal gelatinization torque (C3), starch retrogradation torque (C5–C4), and heating rate (α) of both rice flour doughs, and could maintain the activity of amylase (C3/C4). The 3% polydextrose maintained or even increased the starch breakdown (C3–C4), while 7% polydextrose decreased it. The addition of polydextrose increased the gelatinization rate (β) and maintained the enzymatic degradation rate (γ) of the IP46 rice flour dough but decreased the gelatinization rate and enzymatic degradation rate of the SRN rice flour dough.

A GLM analysis of the thermo-mechanical properties in the early indica rice dough in Table 13 shows that the development time, stable time, protein weakening degree (C1–Cs), C3/C4, C3–C4, α, and γ of the SRN rice flour dough were significantly greater than those of the IP46 rice flour dough, but C3 andC5–C4 were less than those of the IP46 flour dough. Compared with the sample without polydextrose, the addition of two types of polydextrose significantly increased the development time of the rice flour dough, maintained the dough stable time, gelatinization rate, and enzymatic degradation rate, but decreased the gelatinization peak torque (C3), retrogradation torque (C5–C4), and heating rate (α).

### 2.6. Effect of Adding Polydextrose on the Sensory Evaluation of Cooked Early Indica Rice

Table 14 shows that the IP46 rice had a total sensory score of 65.6 and was a paddy that was slightly ineligible for preservation (60–70 score), but the SRN rice had a total sensory score of 80.2 and was a paddy eligible for preservation (≥70 score). Polydextrose addition improved the appearance structure, palatability, and taste score of the cooked IP 46 rice and the taste score of the cooked SRN rice. The total sensory score of the two early indica rice was improved by the polydextrose addition. GLM analysis shows that, compared with the 0% PD addition, both ST and XG polydextrose significantly increased the palatability and taste score of the cooked rice; ST kept the appearance structure of the cooked rice, but XG significantly increased it (Table 15).

### 2.7. Effect of Adding Polydextrose on the Microstructure of Cooked Early Indica Rice

**Figure 2:**
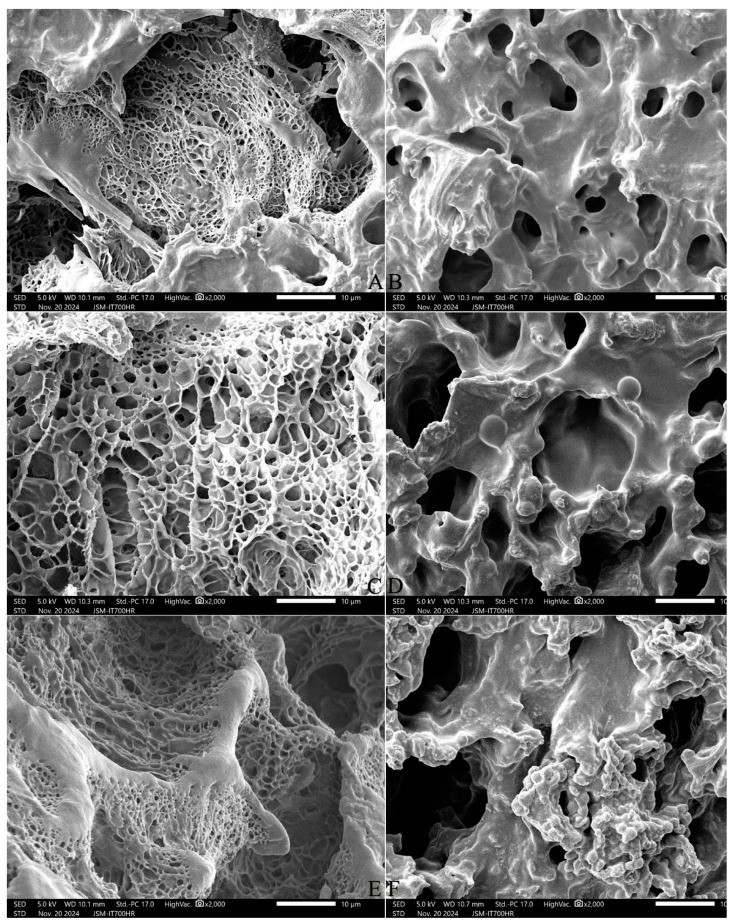
Scanning electron micrographs of the outer surface of cooked rice kernel. Note: (**A**,**C**,**E**) are IP46 variety; (**B**,**D**,**F**) are SRN variety. (**A**,**B**) are CK, (**C**,**D**) are 3% ST addition, (**E**,**F**) are 7% ST addition. All photos are enlarged 2000×.

The surface of the cooked rice kernels all showed different degrees of honeycomb-like pores, and the stale rice of the IP46 variety had abundant filaments (Figure 2). The fresh rice of SRN had relative regular pores and rare filaments in the kernel surface. Polydextrose promoted the entry of water molecules into the surface of the rice kernel and the dissolution of starch, and the honeycomb-like structure was destroyed, resulting in larger and deeper pores. Polydextrose addition dispersed the filamentous aggregate structures and caused the meshes to expand in the IP 46 rice, facilitating water molecules to enter the core of the rice grain.

**Figure 3:**
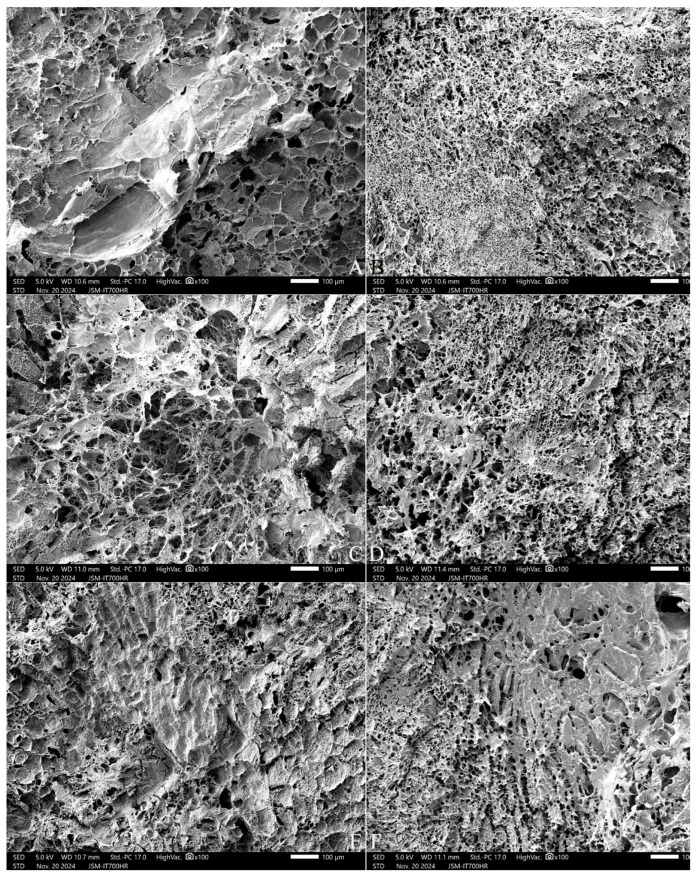
Scanning electron micrographs of the cross-section of a cooked rice kernel. Note: (**A**,**C**,**E**) are IP46 variety; (**B**,**D**,**F**) are SRN variety. (**A**,**B**) are CK, (**C**,**D**) are 3% ST addition, (**E**,**F**) are 7% ST addition. All photos are enlarged 100×.

The cooked rice kernel forms cracks in the cross-section due to the entry of water molecules, and the stale IP46 variety had larger and shallower cracks and more filament aggregates than the fresh SRN variety (Figure 3). The fresh SRN rice had narrower and deeper cracks that were direct to deep endosperm tissue. The entry of water molecules into the rice kernel was promoted by polydextrose, which aggravates the swelling of starch granules, making their internal structure loose and producing obvious depression in the center area of the cross-section.

**Figure 4:**
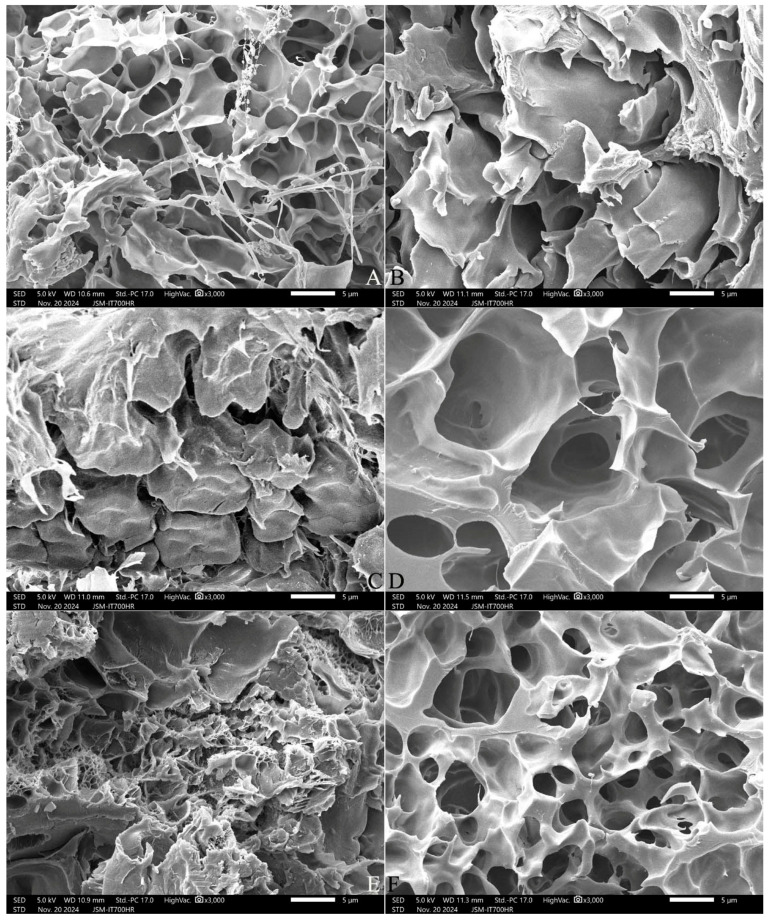
Scanning electron micrographs of the inner layer of cooked rice. Note: (**A**,**C**,**E**) are IP46 variety; (**B**,**D**,**F**) are SRN variety. (**A**,**B**) are CK, (**C**,**D**) are 3% ST addition, (**E**,**F**) are 7% ST addition. All photos are enlarged 3000×.

The cooked kernel of the stale IP46 rice had more shallower pores and filaments, while that of the fresh SRN had larger, more uniform, deeper pores and thicker starch bodies (Figure 4). With the increase in the addition of polydextrose, the honeycomb structure was gradually destroyed, which made the pores larger and the holes shallower, indicating that the recrystallization degree was decreased, and the rice kernels formed a loose and evenly expanded structure, particularly obvious to the fresh SNR rice.

To our knowledge, this study first found that compared with the cooked early indica rice without polydextrose, the cooking time was significantly decreased and the gruel solids loss was significantly increased with the increase in polydextrose addition. Some scholars believe that a significant decrease in the breakdown viscosity of the RVA curve in the pure rice starch/polydextrose system is because polydextrose might reduce the leakage of amylose from the rice starch crystal particles and improve the stability of the rice starch paste [15]. In the present study, the breakdown viscosity in the rice flour/polydextrose system was significantly decreased, but the gruel solids loss in the cooking test was increased, indicating that the addition of polydextrose did not prevent the leakage of amylose from the starch granules in early indica rice, but accelerated the rapid disappearance of the hard core in early indica rice during the cooking process, thus shortening the cooking time. We added 1.0% MC ST and 4.7% MC XG to the cooking rice due to their different humectant performance, with the 1.0% MC ST having a higher hygroscopicity than the 4.7% MC XG. Our recent study showed that the hygroscopicity of amorphous-phase polydextrose powder depends on the molecular weight of the adsorption site polymer; the smaller the molecular of the adsorption site polymer, the lower the water content of the adsorbed monomolecular layer, and the stronger the hygroscopic performance of the sample [18]. GLM analysis showed that XG had a higher capability in decreasing the rice cooking time and the aging of the retrograded rice flour paste, and in increasing the appearance structure and taste score of the cooked rice than ST; ST was better in decreasing the gelatinization enthalpy of the rice flour paste and the setback torque of the rice dough than XG. These distinct differences might be due to the polymer molecular weight and conformation in water.

Leelayuthsoontorn and Thipayarat [19] cooked Jasmine rice under an elevated temperature and pressure to produce a softer texture. Compared with the newly harvested SRN rice, the longer cooking time and the more difficult polydextrose-modified cooking hardness in the IP 46 rice might be due to its biochemical components like higher contents of amylose and protein (Table 14) and the 2.5-year storage time in a storage warehouse. GLM analysis showed that polydextrose addition significantly decreased the hardness of the cooked early indica rice, and increased the gruel solids loss. SEM showed that the stale rice of the IP46 variety had abundant filaments in the kernel surface, but the fresh rice of SRN had relative regular pores and rare filaments (Figure 2). Polydextrose addition dispersed the filamentous aggregate structures and caused the meshes to expand in the IP 46 rice, facilitating water molecules to enter the core of a rice grain. The leachate of cooked rice kernels with polydextrose addition needs further study in stale and fresh rice.

In the starch/polydextrose mixtures, the peak viscosity and final viscosity were decreased in the rice starch/polydextrose mixture with the increase in polydextrose addition [15] but kept unchanged in the corn starch/polydextrose mixture [20]. Both studies clearly showed a 5–20% polydextrose addition led to the decrease in the setback viscosity of the pure cereal starch gels. In the present study, the peak viscosity, final viscosity, and setback viscosity in the early indica rice flour/polydextrose system were decreased with the increase in a3–10% polydextrose addition. This suggests that polydextrose can weaken starch granules and improve cooking stability in early indica rice. The surface of the cooked rice kernel showed different degrees of honeycomb-like pores, while many hydroxyl groups in polydextrose molecules carried water molecules in hydrogen bond form and promoted the entry of water molecules into the surface of the rice kernel and the dissolution of starch and protein through forming new hydrogen bonds; thus, the honeycomb-like structure was destroyed, resulting in larger and deeper pores. The changes in the amorphous regions and crystalline regions of rice starch and the rice protein secondary structure should be further studied by Fourier transform infrared spectroscopy (FTIR). In addition, Jing et al. [21] figured out that 1–3% polydextrose addition could increase the hardness and chewiness texture of steamed bread whilst 5–9% polydextrose addition maintained it. GLM analysis in the present study showed that 3–10% polydextrose addition decreased the hardness and chewiness texture of cooked early indica rice, similar to the results of Schirmer et al. [22], in which 4.4–22% polydextrose addition decreased the firmness of pound cake with the increase in polydextrose concentration.

Rice products are prone to different degrees of quality deterioration during refrigeration, which is considered to be caused by the retrogradation of gelatinized rice starch at low temperatures [23]. Therefore, the addition of sugars, hydrophilic colloids, emulsifiers, and other ways is mainly to inhibit the retrogradation of rice starch [24]. In the present study, the high water solubility and good stability of polydextrose were used to analyze the effect of polydextrose addition on the gel aging characteristics of early indica rice. With the increase in the polydextrose addition concentration, the honeycomb structure of the cooked rice kernels was gradually destroyed, the pores became larger, and the holes became shallower, indicating that the starch recrystallization degree was decreased, and the cooked rice kernels formed a soft and even structure. This is consistent with the results of Chang et al. [15] who added polydextrose to inhibit the gel aging of pure rice starch. The present study suggests that polydextrose could inhibit the aging of cooked early indica rice.

## 3. Conclusions

Double-season paddy planting is one of the means to solve the world’s food shortage, and this study proposes adding polydextrose to improve the problem of the hard texture of cooked early indica rice. Polydextrose has high water solubility and good stability, which significantly reduced the cooking time of early indica rice and increased the gruel solids loss. Two types of polydextrose addition equally reduced the hardness, adhesive force, adhesiveness, cohesiveness, gumminess, and chewiness of the cooked early indica rice. They also equally significantly reduced the peak viscosity, trough viscosity, breakdown viscosity, final viscosity, and setback viscosity of early indica rice paste, and significantly increased the pasting temperature. The addition of two polydextrose significantly increased the gelatinization temperature of rice flour measured by DSC and significantly reduced the gelatinization enthalpy and conclusion temperature. They significantly increased the dough development time of early indica rice flour measured by a Mixolab, but decreased the maximum gelatinization torque, starch breakdown and setback torque, and heating rate. SEM observations confirm that polydextrose promoted the entry of water into the surface of the rice kernels, exacerbated the swelling of starch granules and the dissolution of starch, and the honeycomb structure was gradually destroyed, resulting in larger pores and the formation of soft, evenly swollen cooked rice kernels. A 3–10% polydextrose addition had an effect on improving the texture of cooked rice milled from fresh and aged early indica paddies. The further work is to qualitatively determine the leachate of cooked rice kernels after polydextrose addition, and the changes in the amorphous regions and crystalline regions of the rice starch and rice protein secondary structure.

## 4. Materials and Methods

### 4.1. Materials

Two varieties of early indica rice were sampled from Zhongshan Grain Reserve, Guangdong City, China. The sample of the IP46 variety was stored in a large warehouse for 2.5 years using regular storage technology, and the Sharuan Nian (SRN) variety was newly harvested in September 2023. After being cleaned and packed in a woven bag and a carton box, they were transported to the laboratory of the Academy of National Food and Strategic Reserves Administration, and then separately packed into No. 11 valve bags (400 × 280 × 0.04 mm of length × width × thickness, Apple Brand, Shanghai China Manufacturing) to be kept in a plastic box (53.5 × 40 × 30 cm of length × width × height) with a cover in a 15.0 °C cold store. The moisture contents (MCs) of the two indica rice samples were 11.12% and 10.67% wet basis, respectively (Table 16). Two kinds of the polydextrose samples (ST and XG) with respective moisture contents of 1.0% and 4.7% were provided by Runloy Biotech (Shanghai) Co., Ltd., Shanghai, China. The early indica rice kernels or flours each had polydextrose added at the ratios of 0%, 3%, 5%, 7%, and 10%, respectively, as the test samples.

The moisture content of the samples was determined by the oven-drying method [25]. Percent of milled rice was determined as described by Gao et al. [26]. Head rice percent and kernel length to width ratio were determined using an Appearance Quality Instrument for rice JMWT (Beijing Dongfu Jiuheng Instrument Technology Co., Ltd. and Satake, Beijing, China) [26]. The taste value of milled rice was determined using a rice taste meter (Dongfu Jiuheng-Satake, Beijing Dongfu Jiuheng Instrument Technology Co., Ltd., China), as described by Tao et al. [27]. Amylose content in milled rice was determined according to GB/T 15683-2008 [28]. Content of protein in milled rice was determined according to ISO 14891-2002 [29]. A rapid N cube nitrogen analyzer (Elementar Rapid, Frankfurt, Germany) was used. The content of free fatty acid (FFA) in rice flours was measured according to the procedure described by GB/T 5510-2011 [30].

### 4.2. Cooking Time, Water Uptake Ratio, and Gruel Solids Loss

We made an optimization to the determination method of the cooking properties of milled rice. An amount of 2 g of the rice sample was added into a hard glass test tube (2.5 cm diameter × 20 cm length) and soaked for 5 min in 20 mL deionized water. The glass test tube was then put into the grid (3.3 cm length × 3.3 cm width × 6.4 cm height) of a test tube rack made of steel wires and cooked in a boiling water bath in an electromagnetic cooker (C21-SDHCB8E455, Zhejiang Supor Co., Ltd., Shaoxing city, China). The test tube rack (16 cm length × 16 cm width × 6.4 cm height) had16 grids. The minimal cooking time was measured by extracting a single rice kernel at 30 s intervals during cooking, placing it on a glass plate, and pressing it using the bottom of a spoon head (3.5 cm length × 2.1 cm width) with a 17.4 cm long handle. The rice was cooked until no white core was seen in any kernels when pressing.

Rice samples (each 2 g) were cooked in 20 mL of deionized water for a minimal cooking time in the boiling water bath. After cooking, the excess water was drained off in a high-speed freezing centrifuge (3–30 K, Sigma-Aldrich Trading Co., Ltd., Shanghai, China) at 4000 rpm for 6 min, and the contents were transferred to a filter paper to remove the surface water. The cooked samples were weighed on an electronic balance (0.0001 g, AL204-IC, Mettler Toledo Technology (China) Co., Ltd., Shanghai, China), and the water uptake ratio was reckoned using the method of Singh et al. [31].

The rice samples (each 2 g) in three parallels were cooked in 20 mL of deionized water for a minimal cooking time in the boiling water bath. The resulting gruel was transferred into glass Petri dishes (15.5 cm diameter and 2.3 cm height). The cooked rice was subjected to repeated deionized water washings to extract the solids adhering to the rice surface. The pooled extracts were completely dried at 110 °C in an electrothermostatic blast oven (DHG 9070A, Blue Sky Laboratory Instrument, Hangzhou, China). The dried solids were weighed on the balance (0.0001 g, AL204-IC, Mettler Toledo), and gruel solids loss was reckoned using the method of Singh et al. [31]. The gruel solids loss suggests the leakage of amylose from starch granules when cooking rice. The cooked rice will be more delicious when more amylose leaks into the cooking water [32]. The test for the determination of the rice cooking time was repeated five times, but the measurements of the water uptake ratio and gruel solids loss were each repeated three times.

### 4.3. Texture Profile of Cooked Rice

The texture of the cooked rice was measured using a texture profile analyzer (CTX, AMETEK Brookfield, Middleboro, MA, USA). The cooked rice was prepared in accordance with GB/T 15682-2008 [33]. The polydextrose was added to 19.5 g of deionized water at the ratio of 0%, 3%, 5%, 7, and 10%, and the mass of rice kernels and polydextrose was 15 g. The ratio of samples to deionized water was 1:1.3. The samples were soaked at room temperature for 30 min and then placed in a boiling water pot for steaming for 40 min. After the heating was stopped, the samples were suffocated for 20 min and then the texture characteristics were measured using a cylindrical P35 probe (height 4 cm, diameter 3.5 cm) of the texture profile analyzer. The parameters were set as follows: the pre-test, test, and post-test speeds were all 2 mm/s, the compression distance was 15 mm, and sensing force was 5 g. The hardness, adhesiveness, springiness, resilience, cohesiveness, gumminess, and chewiness were measured. Hardness cycle is the force required to reach the specified deformation. Adhesiveness is the work performed to overcome the attraction between the surface of a food and the surface of a contacting object. Adhesive force is the force required to pull the probe out of the sample. Resilience is a value that measures the degree to which a deformed sample recovers under the same velocity and pressure conditions that caused the deformation. Cohesiveness indicates the internal cohesive force of the sample, and when the cohesiveness is above the adhesion and the probe is in full contact with the sample, the probe can still remain clean without any sample adhering to it. Springiness (mm) is the height or volume ratio of a deformed sample that recovers to its original condition after the deforming force is removed. Gumminess (hardness × cohesiveness, g) indicates the energy required to break a semisolid sample into a stable state suitable for swallowing. Chewiness (hardness × cohesiveness × springiness, mJ) indicates the energy required to chew a semisolid sample into a stable state suitable for swallowing.

### 4.4. PastingCharacteristics

The RVA—TecMaster (Perten Ruihua Scientific Instrument Beijing Co., Ltd., Beijing, China) was used to measure the pasting profiles of rice flour samples with polydextrose addition according to GB/T24852-2010 [34]. The speed setting of the stirring paddle during the measurement was the following: the initial 10 s at a speed of 960 r/min, then decreased to 160 r/min within 20 s and maintained. The temperature setting during the measurement was firstly held at 50 °C for 1 min, then increased to 95 °C within 3.7 min and maintained for 2.5 min, finally decreased to 50 °C within 2.8 min and held for 2 min. The RVA-specific TCW software (TCW3 version) was used to analyze the viscosity curve of rice flour pasting, and the viscosity unit was expressed in cp.

### 4.5. Thermal Properties

The thermal properties of the samples of rice flour with polydextrose addition were determined using a differential scanning calorimeter (DSC 214, Netzsch GmbH, Freistaat Bayern, Germany). Rice flour and polydextrose were mixed uniformly according to the addition of 0%, 3%, 5%, 7%, and 10% polydextrose, and 5.0 mg of the sample was weighed and placed in an aluminum flat crucible, and 10 μL of distilled water was added. After sealing, it was placed in a 4 °C environment for overnight equilibration and used for the test. Each sample was measured in triplicate. During the test, a sealed empty crucible was used as a reference. The differential scanning calorimeter (DSC) scanning temperature range was 20 °C to 110 °C, and the heating rate was 10 °C/min. Protective gas 1 and protective 2 were both nitrogen. After the measurement was completed, the results were analyzed using the data analysis software provided with the DSC 214 instrument, and the gelatinization parameters of rice starch were obtained: gelatinization enthalpy, gelatinization peak temperature (*T*_p_), gelatinization onset temperature (*T*_o_), gelatinization conclusion temperature (*T*_c_), gelatinization width, and height.

The samples after gelatinization in the sealed crucible were placed into 24-well plates with covers (12.5 × 8 × 2 cm of length × width × height) in a 4 °C refrigerator for 21 days then measured for retrogradation, and the results were analyzed using the data analysis software provided with the DSC 214 instrument.

### 4.6. Thermo-Mechanical Properties

A Mixolab was used for measuring the thermo-mechanical properties of rice flour dough. The mixing and pasting behavior of early rice flour dough was determined using a Mixolab (Chopin Technologies, Tripette et Renaud, Paris, France), according to Rosell et al. [35]. For an assay under a constant hydration, 56–61 g of rice flour was added into the Mixolab bowl. A dough (90 g) with 60% water level (14% moisture basis) was assessed. The temperature of the water tank and the initial mixing was 30 °C. The mixing speed was 80 rpm/min during the entire analysis. The initial mixing was fulfilled at 30 °C for 8 min. The temperature was then ascended to 90 °C within 15 min at a speed of 4 °C/min. After 7 min at 90 °C, the temperature was reduced to 50 °C within 10 min at a speed of 4 °C/min, and kept for 5 min. Total time of an assay was 45 min. The interest parameters were protein weakness at the constant-temperature phase (C1–Cs), starch breakdown (C3–C4), amylase activity (C3/C4), and starch setback (C5–C4), as well as dough development time (DDT) and stability time (DST). Protein weakening (Nm) is the difference between the maximal torque (C1) at 30 °C and the torque (Cs) at the end of the remaining time at 30 °C. Amylase activity is a ratio of the maximal gelatinization torque (C3) during the heating period to the torque (C4) after keeping at 90 °C. Starch breakdown (Nm) is the difference between C3 and C4. Starch setback (Nm) is the difference between C5 torque produced after cooling at 50 °C and C4 torque after the heating period. The maximal gelatinization torque (C3), heating rate (α, −0.01 Nm/min), gelatinization rate (β, Nm/min), and enzymatic degradation rate (γ, −0.01 Nm/min) were also used.

### 4.7. Sensory Evaluation of Cooked Rice

The cooked rice was evaluated using the method of GB/T 15682-2008 [33]. Eight evaluators (four male and four female) aged between 18 and 40 were selected to observe, taste, and evaluate the cooked rice, including smell, appearance structure, palatability, and taste, as well as cool rice texture, and total score.

### 4.8. Scanning Electron Microscopy (SEM)

The cooked rice samples were cooled to room temperature and stored in a −20 °C refrigerator. Before the experiment, the samples were placed in a freeze dryer for lyophilization. The dried samples were fixed on the sample stage with double-sided conductive glue and then sputtered with gold using a vacuum gold particle sprayer (JEC-3000FC type ion sputtering instrument, Japan Electronics Co., Ltd., Tokyo, Japan). The samples were then fixed on the stage of a scanning electron microscope (JSM-IT 700HR type, Japan Electronics Co., Ltd.) and observed at an accelerating voltage of 25 kV with a magnification of 100 to 2000 times.

### 4.9. Data Analysis

The results were statistically analyzed with SPSS software (Version 17.0, SPSS Incorporated [36]). For the control (CK) and four polydextrose-treated samples within each indica rice variety, one-way ANOVA method and Duncan’s new multiple-range test were, respectively, adopted to compare multiple and pairs of mean values. To observe the effect of the rice variety and polydextrose treatment, the General Linear Model Univariate method was chosen to compare the means by the LSD test. Statistical significance was given at *p* <0.05 level.

## Figures and Tables

**Table 1:** Effect of adding polydextrose on the cooking characteristics of early indica rice.

Variety	PD	Cooking Time (min)	Water Uptake Ratio	Gruel Solids Loss (mg/g)
IP46	CK	24.12 ± 0.09 ^a^	3.21 ± 0.13 ^a^	38.96 ± 6.54 ^e^
	3%ST	24.06 ± 0.05 ^a^	3.18 ± 0.20 ^ab^	58.54 ± 5.63 ^d^
	5%ST	24.02 ± 0.13 ^ab^	3.15 ± 0.12 ^ab^	72.48 ± 2.28 ^c^
	7%ST	23.97 ± 0.08 ^ab^	3.16 ± 0.19 ^ab^	83.09 ± 0.88 ^b^
	10%ST	23.56 ± 0.14 ^c^	3.03 ± 0.07 ^b^	108.70 ± 3.13 ^a^
	3%XG	24.10 ± 0.04 ^a^	3.16 ± 0.06 ^ab^	57.68 ± 0.96 ^d^
	5%XG	23.88± 0.06 ^b^	3.29 ± 0.13 ^a^	72.03± 1.57 ^c^
	7%XG	23.73± 0.07 ^c^	3.20 ± 0.11 ^ab^	85.51± 2.38 ^b^
	10%XG	23.36 ± 0.01 ^d^	3.27 ± 0.05 ^a^	106.48 ± 3.91 ^a^
SRN	CK	18.87± 0.25 ^a^	3.26 ± 0.15 ^ab^	77.02± 6.85 ^g^
	3%ST	18.63 ± 0.26 ^ab^	3.39 ± 0.08 ^a^	92.65± 7.53 ^e^
	5%ST	18.50± 0.25 ^ab^	3.40± 0.34 ^ab^	102.19± 1.80 ^d^
	7%ST	18.37± 0.13 ^b^	3.21 ± 0.12 ^ab^	138.63 ± 4.30 ^b^
	10%ST	18.38 ± 0.11 ^b^	3.32± 0.02 ^a^	141.37 ± 7.55 ^ab^
	3%XG	18.67 ± 0.04 ^a^	2.83 ± 0.09 ^c^	95.75± 1.99 ^f^
	5%XG	18.12 ± 0.04 ^c^	3.19 ± 0.09 ^b^	104.92 ± 8.12 ^d^
	7%XG	17.83 ± 0.03 ^d^	3.26± 0.10 ^ab^	119.73± 4.85 ^c^
	10%XG	17.59 ± 0.04 ^e^	2.95± 0.05 ^c^	147.68 ± 4.18 ^a^

Note: SRN, Sharuan Nian; PD, polydextrose; CK, the sample without PD. All the data are expressed as mean ± SD, number of repetitions—*n* = 5 for cooking time and *n* = 3 for water uptake ratio and gruel solids loss. Different superscript letters indicate the significant differences (*p* < 0.05) within the column for each early indica variety.

**Table 2:** Generalized linear model (GLM) analysis of the cooking properties of early indica rice.

Factors	Level	Cooking Time (min)	Water Uptake Ratio	Gruel Solids Loss (mg/g)
Rice	IP46	23.89 ± 0.04 ^a^	3.19 ± 0.03 ^ab^	72.24 ± 1.10 ^g^
variety	SRN	18.35 ± 0.04 ^h^	3.20 ± 0.03 ^ab^	109.69 ± 1.10 ^b^
PD type	ST	21.25 ± 0.04 ^d^	3.23 ± 0.03 ^a^	91.36 ± 1.10 ^d^
	XG	21.03 ± 0.04 ^f^	3.16 ± 0.03 ^b^	90.58 ± 1.10 ^de^
PD	0	21.49 ± 0.06 ^b^	3.24 ± 0.05 ^ab^	57.99 ± 1.73 ^h^
addition	3	21.37 ± 0.06 ^c^	3.14 ± 0.05 ^b^	76.15 ± 1.73 ^f^
(%)	5	21.13 ± 0.06 ^e^	3.26 ± 0.05 ^a^	87.91 ± 1.73 ^e^
	7	20.97 ± 0.06 ^f^	3.21 ± 0.05 ^ab^	106.74 ± 1.73 ^c^
	10	20.72 ± 0.06 ^g^	3.14 ± 0.05 ^b^	126.06 ± 1.73 ^a^

Note: SRN, Sharuan Nian; PD, polydextrose. All the data are expressed as mean ± SD, number of repetitions—*n* = 5 for cooking time and *n* = 3 for water uptake ratio and gruel solids loss. Different superscript letters indicate the significant differences (*p* < 0.05) within the column.

**Table 3:** Effect of adding polydextrose on the texture of cooked early indica rice.

Rice Variety	PD Addition	Hardness (g)	Adhesive Force (g)	Adhesiveness (mJ)	Resilience
IP46	CK	2246 ± 257 ^bc^	27.8 ± 11.7 ^abc^	0.81 ± 0.44 ^a^	0.17 ± 0.02 ^a^
	3%ST	2436 ± 529 ^abc^	19.5 ± 8.7 ^bc^	0.43 ± 0.21 ^abc^	0.16 ± 0.01 ^a^
	5%ST	2976 ± 651 ^abc^	33.7 ± 13.0 ^ab^	0.76 ± 0.33 ^ab^	0.16 ± 0.01 ^a^
	7%ST	2590± 335 ^abc^	22.7 ± 10.0 ^abc^	0.53± 0.23 ^abc^	0.15± 0.01 ^a^
	10%ST	3220 ± 510 ^a^	36.3 ± 12.6 ^ab^	0.93± 0.42 ^a^	0.16 ± 0.02 ^a^
	3%XG	2040 ± 331 ^c^	12.6 ± 3.8 ^c^	0.24 ± 0.06 ^c^	0.16± 0.01 ^a^
	5%XG	2916 ± 166 ^a^	35.1 ± 3.1 ^a^	0.80± 0.14 ^a^	0.15± 0.01 ^a^
	7%XG	2260 ± 330 ^bc^	17.5 ± 9.2 ^bc^	0.33 ± 0.18 ^bc^	0.17 ± 0.02 ^a^
	10%XG	2853 ± 354 ^ab^	26.7 ± 9.2 ^ab^	0.66± 0.24 ^ab^	0.15± 0.01 ^a^
SRN	CK	4578 ± 809 ^a^	348.9 ± 50.0 ^a^	24.49 ± 8.44 ^a^	0.09 ± 0.01 ^a^
	3%ST	3778 ± 272 ^a^	271.2 ± 35.1 ^ab^	9.14± 2.56 ^bc^	0.09 ± 0.02 ^a^
	5%ST	3732 ± 165 ^a^	254.8 ± 9.8 ^b^	7.68 ± 0.37 ^b^	0.09 ± 0.01 ^a^
	7%ST	3550 ± 301 ^abc^	246.4 ± 59.5 ^abc^	7.46 ± 2.66 ^bc^	0.08 ± 0.01 ^a^
	10%ST	2999± 314 ^c^	192.7 ± 24.0 ^c^	5.28± 1.30 ^c^	0.11 ± 0.03 ^a^
	3%XG	3785 ± 200 ^a^	262.1 ± 54.1 ^abc^	8.48 ± 2.33 ^bc^	0.10 ± 0.01 ^a^
	5%XG	3601 ± 127 ^b^	279.7 ± 22.6 ^a^	9.97 ± 1.23 ^b^	0.09 ± 0.01 ^a^
	7%XG	3352 ± 140 ^bc^	207.4 ± 17.1 ^c^	5.94 ± 0.95 ^c^	0.11 ± 0.02 ^a^
	10%XG	3256 ± 715 ^abc^	236.5± 72.1 ^abc^	7.12 ± 3.06 ^c^	0.11 ± 0.02 ^a^
Rice variety	PD addition	Cohesiveness	Springiness (mm)	Gumminess (g)	Chewiness (mJ)
IP46	CK	0.36 ± 0.02 ^a^	8.73 ± 1.02 ^a^	818.2 ± 144.6 ^abc^	70.0 ± 14.5 ^ab^
	3%ST	0.34 ± 0.02 ^a^	9.77 ± 1.10 ^a^	819.4 ± 166.3 ^abc^	79.8 ± 25.0 ^ab^
	5%ST	0.35 ± 0.02 ^a^	11.32 ± 2.05 ^a^	1051.8 ± 248.2 ^abc^	120.2 ± 43.0 ^ab^
	7%ST	0.34 ± 0.01 ^a^	9.77 ± 1.71 ^a^	881.2 ± 116.2 ^abc^	85.5± 24.0 ^ab^
	10%ST	0.32 ± 0.02 ^a^	10.93 ± 1.42 ^a^	1022.7 ± 131.5 ^a^	111.0± 29.3 ^a^
	3%XG	0.36 ± 0.03 ^a^	8.94 ± 1.05 ^a^	720.5 ± 83.3 ^c^	63.8 ± 15.4 ^b^
	5%XG	0.34 ± 0.02 ^a^	10.16 ± 1.56 ^a^	984.1 ± 74.0 ^a^	98.6 ± 20.2 ^ab^
	7%XG	0.34 ± 0.02 ^a^	8.17 ± 1.35 ^a^	770.7 ± 77.7 ^bc^	62.5 ± 16.6 ^b^
	10%XG	0.34 ± 0.03 ^a^	9.73 ± 1.14 ^a^	952.6 ± 125.1 ^ab^	91.6 ± 20.3 ^ab^
SRN	CK	0.35 ± 0.03 ^a^	13.46 ± 2.45 ^ab^	1588.8 ± 304.8 ^a^	215.0 ± 83.1 ^a^
	3%ST	0.27 ± 0.01 ^b^	10.98 ± 1.15 ^bc^	1006.3 ± 79.2 ^bc^	108.7 ± 17.5 ^bc^
	5%ST	0.28 ± 0.02 ^b^	12.56 ± 0.94 ^ab^	1026.3 ± 87.4 ^b^	126.8 ± 19.0 ^ab^
	7%ST	0.30 ± 0.04 ^ab^	12.67 ± 0.43 ^a^	1030.1 ± 68.4 ^b^	128.1 ± 11.0 ^ab^
	10%ST	0.29 ± 0.01 ^b^	10.92 ± 0.86 ^b^	852.7 ± 73.9 ^d^	91.6± 14.1 ^c^
	3%XG	0.27 ± 0.03 ^b^	11.33 ± 1.40 ^abc^	1010.2 ± 51.4 ^b^	111.9 ± 11.6 ^bc^
	5%XG	0.27± 0.01 ^b^	10.91 ± 0.83 ^bc^	986.1 ± 54.2 ^bc^	105.7 ± 13.4 ^bc^
	7%XG	0.27 ± 0.01 ^b^	10.63 ± 0.40 ^c^	895.0 ± 43.7 ^cd^	93.4 ± 7.9 ^c^
	10%XG	0.28 ± 0.01 ^b^	10.46 ± 2.00 ^abc^	902.2 ± 180.2 ^bcd^	94.4 ± 31.6 ^bc^

Note: SRN, Sharuan Nian; PD, polydextrose; CK, the sample without PD. All the data are expressed as mean ± SD, number of repetitions—*n* = 3. Different superscript letters indicate the significant differences (*p* < 0.05) within the column for each early indica rice variety.

**Table 4:** The GLM analysis of the texture of early indica rice.

Factors	Level	Hardness (g)	Adhesive Force (g)	Adhesiveness (mJ)	Resilience
Rice	IP46	2578 ± 90 ^f^	25.9 ± 6.2 ^d^	0.63 ± 0.71 ^d^	0.159 ± 0.003 ^a^
variety	SRN	3721 ± 90 ^a^	264.8 ± 6.2 ^a^	11.00 ± 0.71 ^a^	0.092 ± 0.003 ^d^
PD	ST	3211 ± 90 ^bcd^	145.4 ± 6.2 ^b^	5.75 ± 0.71 ^b^	0.123 ± 0.003 ^bc^
type	XG	3089 ± 90 ^cde^	145.4 ± 6.2 ^b^	5.88 ± 0.71 ^b^	0.128 ± 0.003 ^b^
PD	0	3412 ± 142 ^b^	188.3 ± 9.9 ^a^	12.65 ± 1.12 ^a^	0.128 ± 0.004 ^b^
(%)	3	3010 ± 142 ^de^	141.3 ± 9.9 ^bc^	4.57 ± 1.12 ^bc^	0.124 ± 0.004 ^bc^
	5	3306 ± 142 ^bc^	150.8 ± 9.9 ^b^	4.80 ± 1.12 ^bc^	0.119 ± 0.004 ^c^
	7	2938 ± 142 ^e^	123.5 ± 9.9 ^c^	3.56 ± 1.12 ^c^	0.127 ± 0.004 ^bc^
	10	3082 ± 142 ^cde^	123.1 ± 9.9 ^c^	3.49 ± 1.12 ^c^	0.129 ± 0.004 ^b^
Factors	Level	Cohesiveness	Springiness (mm)	Gumminess (g)	Chewiness (mJ)
Rice	IP46	0.345 ± 0.004 ^a^	9.63 ± 0.25 ^e^	884 ± 34 ^f^	85.3 ± 6.6 ^e^
variety	SRN	0.290 ± 0.004 ^e^	11.74 ± 0.25 ^a^	1089 ± 34 ^b^	129.0 ± 6.6 ^ab^
PD	ST	0.318 ± 0.004 ^b^	11.11 ± 0.25 ^b^	1010 ± 34 ^c^	113.7 ± 6.6 ^c^
type	XG	0.317 ± 0.004 ^bc^	10.25 ± 0.25 ^d^	963 ± 34 ^cd^	100.7 ± 6.6 ^bcd^
PD	0	0.354 ± 0.006 ^a^	11.09 ± 0.39 ^bc^	1204 ± 53 ^a^	142.5 ± 10.4 ^a^
(%)	3	0.307 ± 0.006 ^cd^	10.26 ± 0.39 ^de^	889 ± 53 ^df^	91.1 ± 10.4 ^de^
	5	0.309 ± 0.006 ^bcd^	11.24 ± 0.39 ^ab^	1012 ± 53 ^bc^	112.8 ± 10.4 ^bcd^
	7	0.311 ± 0.006 ^bcd^	10.31 ± 0.39 ^cd^	894 ± 53 ^df^	92.4 ± 10.4 ^de^
	10	0.304 ± 0.006 ^d^	10.51 ± 0.39 ^bcd^	933 ± 53 ^cdf^	97.1 ± 10.4 ^cde^

Note: SRN, Sharuan Nian; PD, polydextrose. All the data are expressed as mean ± SD, number of repetitions—*n* = 3. Different superscript letters indicate the significant differences (*p* < 0.05) within the column.

**Table 5:** Effect of adding polydextrose on the pasting property of early indica rice flours.

Rice Variety	PD Addition	Peak Viscosity (cp)	Trough Viscosity (cp)	Breakdown Viscosity (cp)	Final Viscosity (cp)
IP46	CK	3743 ± 31 ^a^	3191 ± 21 ^a^	551 ± 15 ^a^	5659 ± 53 ^a^
	3%ST	3566 ± 52 ^b^	3090 ± 40 ^b^	476 ± 13 ^b^	5497 ± 63 ^b^
	5%ST	3143 ± 20 ^d^	2814 ± 28 ^d^	328 ± 14 ^d^	5066 ± 23 ^d^
	7%ST	2802 ± 23 ^g^	2559 ± 15 ^f^	244 ± 11 ^e^	4610 ± 28 ^f^
	10%ST	2444 ± 45 ^h^	2312 ± 40 ^g^	132 ± 6 ^f^	4130 ± 17 ^g^
	3%XG	3404 ± 19 ^c^	3014± 23 ^c^	390 ± 13 ^c^	5374 ± 26 ^c^
	5%XG	3079 ± 30 ^e^	2759 ± 40 ^d^	320 ± 35 ^d^	4989 ± 61 ^d^
	7%XG	2912 ± 40 ^f^	2649 ± 45 ^e^	263 ± 9 ^e^	4787 ± 38 ^e^
	10%XG	2511 ± 134 ^h^	2353 ± 99 ^g^	157 ± 35 ^f^	4234 ± 197 ^g^
SRN	CK	5233 ± 63 ^a^	2552 ± 64 ^a^	2680 ± 49 ^a^	3917 ± 43 ^a^
	3%ST	4846 ± 255 ^b^	2557 ± 101 ^a^	2289 ± 157 ^b^	3792 ± 121 ^ab^
	5%ST	4507 ± 167 ^bcd^	2503 ± 127 ^ab^	2005 ± 43 ^c^	3710 ± 65 ^bc^
	7%ST	4224 ± 84 ^d^	2451 ± 202 ^ab^	1773 ± 157 ^de^	3596 ± 175 ^bcde^
	10%ST	3857 ± 34 ^e^	2403 ± 109 ^ab^	1455 ± 116 ^f^	3494 ± 122 ^de^
	3%XG	4569 ± 48 ^bc^	2458 ± 148 ^ab^	2111 ± 151 ^b^	3753 ± 155 ^abcd^
	5%XG	4461 ± 73 ^c^	2408 ± 112 ^ab^	2053 ± 40 ^c^	3585 ± 72 ^cde^
	7%XG	4266 ± 84 ^d^	2343 ± 51 ^b^	1923 ± 34 ^d^	3542 ± 6 ^e^
	10%XG	3931 ± 21 ^e^	2418 ± 160 ^ab^	1513 ± 182 ^ef^	3515 ± 137 ^cde^
Rice variety	PD addition	Setback viscosity (cp)	Peak time (min)	Pasting temp. (°C)	
IP46	CK	2468 ± 32 ^a^	5.80 ± 0.00 ^f^	87.17 ± 0.03 ^f^	
	3%ST	2407 ± 24 ^b^	5.87 ± 0.00 ^e^	88.00 ± 0.00 ^c^	
	5%ST	2252 ± 37 ^d^	5.95 ± 0.04 ^d^	89.08 ± 0.49 ^cd^	
	7%ST	2051 ± 39 ^f^	6.11± 0.03 ^b^	89.83 ± 0.49 ^bcd^	
	10%ST	1818 ± 27 ^g^	6.25 ± 0.04 ^a^	91.18 ± 0.03 ^a^	
	3%XG	2360 ± 7 ^c^	5.93 ± 0.00 ^d^	88.48 ± 0.46 ^d^	
	5%XG	2230 ± 49 ^d^	6.00 ± 0.07 ^cd^	89.00 ± 0.52 ^d^	
	7%XG	2138 ± 10 ^e^	6.05 ± 0.04 ^bc^	89.65 ± 0.05 ^b^	
	10%XG	1880 ± 99 ^g^	6.16 ± 0.08 ^ab^	90.45 ± 0.75 ^ab^	
SRN	CK	1365 ± 42 ^a^	5.69 ± 0.03 ^d^	74.20 ± 0.05 ^d^	
	3%ST	1235 ± 53 ^b^	5.80± 0.13 ^bcd^	74.77 ± 0.45 ^c^	
	5%ST	1207 ± 63 ^c^	5.91 ± 0.03 ^b^	75.57 ± 0.45 ^abc^	
	7%ST	1144 ± 40 ^c^	6.02± 0.16 ^abc^	76.15 ± 0.52 ^ab^	
	10%ST	1091 ± 13 ^d^	6.14 ± 0.12 ^a^	76.33 ± 0.51 ^a^	
	3%XG	1295 ± 9 ^b^	5.89 ± 0.10 ^bc^	75.03 ± 0.03 ^c^	
	5%XG	1177 ± 41 ^c^	5.82 ± 0.04 ^c^	75.32 ± 0.51 ^bc^	
	7%XG	1199 ± 46 ^c^	5.87 ± 0.07 ^bc^	75.52 ± 0.49 ^abc^	
	10%XG	1097 ± 31 ^d^	6.09 ± 0.21 ^ab^	76.15 ± 0.52 ^ab^	

Note: SRN, Sharuan Nian; PD, polydextrose; CK, the sample without PD. All the data are expressed as mean ± SD, number of repetitions—*n* = 3. Different superscript letters indicate the significant differences (*p* < 0.05) within the column for each early indica rice variety.

**Table 6:** The GLM analysis of pasting properties of early indica rice flours.

Factors	Level	Peak Viscosity (cp)	Trough Viscosity (cp)	Breakdown Viscosity (cp)	Final Viscosity (cp)
Rice	IP46	3135 ± 20 ^f^	2793 ± 29 ^b^	341 ± 21 ^g^	5000 ± 41 ^a^
variety	SRN	4513 ± 20 ^a^	2464 ± 29 ^d^	2048 ± 21 ^a^	3682 ± 41 ^g^
PD type	ST	3836 ± 20 ^c^	2643 ± 29 ^c^	1193 ± 21 ^d^	4347 ± 41 ^d^
	XG	3811 ± 20 ^c^	2614 ± 29 ^c^	1196 ± 21 ^d^	4335 ± 41 ^d^
PD	0	4488 ± 31 ^a^	2872 ± 46 ^a^	1616 ± 46 ^b^	4788 ± 65 ^b^
(%)	3	4096 ± 31 ^b^	2779 ± 46 ^ab^	1316 ± 46 ^c^	4604 ± 65 ^c^
	5	3797 ± 31 ^c^	2621 ± 46 ^c^	1177 ± 46 ^d^	4337 ± 65 ^d^
	7	3551 ± 31 ^d^	2500 ± 46 ^d^	1051 ± 46 ^e^	4134 ± 65 ^e^
	10	3186 ± 31 ^e^	2372 ± 46 ^e^	814 ± 46 ^f^	3843 ± 65 ^f^
Factors	Level	Setback viscosity(cp)	Peak time (min)	Pasting temp. (°C)	
Rice	IP46	2207 ± 15 ^a^	5.99 ± 0.02 ^a^	89.00 ± 0.09 ^a^	
variety	SRN	1218 ± 15 ^g^	5.89 ± 0.02 ^c^	75.32 ± 0.09 ^g^	
PD type	ST	1704 ± 15 ^d^	5.95 ± 0.02 ^b^	82.23 ± 0.09 ^d^	
	XG	1721 ± 15 ^d^	5.93 ± 0.02 ^b^	82.09 ± 0.09 ^d^	
PD	0	1916 ± 24 ^b^	5.75 ± 0.02 ^d^	80.68 ± 0.14 ^f^	
(%)	3	1824 ± 24 ^c^	5.87 ± 0.02 ^c^	81.57 ± 0.14 ^e^	
	5	1717 ± 24 ^d^	5.92 ± 0.02 ^b^	82.24 ± 0.14 ^d^	
	7	1633 ± 24 ^e^	6.01 ± 0.02 ^a^	82.79 ± 0.14 ^c^	
	10	1472 ± 24 ^f^	6.16 ± 0.02 ^a^	83.53 ± 0.14 ^b^	

Note: SRN, Sharuan Nian; PD, polydextrose. All the data are expressed as mean ± SD, number of repetitions—*n* = 3. Different superscript letters indicate the significant differences (*p* < 0.05) within the column.

**Table 7:** Effect of adding polydextrose on thermal property of early indica rice flours determined on day 0.

Rice Variety	PD Addition	Δ*H* (J/g)	*T*_o_ (°C)	*T*_p_ (°C)	*T*_c_ (°C)	Peak Width (°C)	Peak Height (0.01 mW/mg)
IP46	CK	15.04 ± 1.93 ^a^	70.77 ± 1.33 ^ab^	79.77 ± 0.06 ^d^	96.37 ± 6.90 ^a^	9.90 ± 0.40 ^a^	16.63 ± 1.02 ^ab^
	3%ST	13.50 ± 1.99 ^a^	70.80 ± 0.85 ^ab^	80.00 ± 0.10 ^b^	92.57 ± 1.08 ^a^	10.03 ± 0.78 ^a^	14.57 ± 1.12 ^bcd^
	5%ST	13.16 ± 0.24 ^a^	71.10 ± 0.57 ^ab^	79.95 ± 0.07 ^bc^	92.45 ± 0.49 ^a^	9.95 ± 0.35 ^a^	14.86 ± 0.16 ^d^
	7%ST	13.97 ± 0.69 ^a^	70.20 ± 0.46 ^ab^	80.33 ± 0.06 ^a^	92.00 ± 0.46 ^a^	9.87 ± 0.35 ^a^	15.47 ± 0.49 ^abcd^
	10%ST	12.50 ± 1.98 ^a^	70.73 ± 0.45 ^ab^	80.73 ± 0.76 ^a^	91.40 ± 1.21 ^a^	9.53 ± 0.55 ^a^	14.40 ± 0.90 ^cd^
	3%XG	15.48 ± 0.89 ^a^	70.23 ± 0.32 ^b^	79.60 ± 0.20 ^d^	92.13 ± 0.25 ^a^	9.90 ± 0.10 ^a^	16.11 ± 0.23 ^a^
	5%XG	14.52 ± 0.64 ^a^	70.47 ± 0.35 ^ab^	79.83 ± 0.06 ^c^	92.00 ± 0.20 ^a^	9.83 ± 0.12 ^a^	15.55 ± 0.09 ^b^
	7%XG	14.64 ± 0.31 ^a^	70.70 ± 0.10 ^a^	80.37 ± 0.29 ^a^	91.87 ± 0.67 ^a^	9.73 ± 0.25 ^a^	15.58 ± 0.28 ^bc^
	10%XG	15.53 ± 2.47 ^a^	70.63 ± 0.32 ^ab^	80.47 ± 0.12 ^a^	91.83 ± 0.47 ^a^	9.60 ± 0.30 ^a^	17.04 ± 1.96 ^abc^
SRN	CK	14.95 ± 1.26 ^a^	64.90 ± 0.17 ^e^	70.13 ± 0.21 ^e^	79.67 ± 0.49 ^ab^	6.83 ± 0.2^1 a^	19.80 ± 0.80 ^ab^
	3%ST	14.42 ± 1.17 ^a^	65.07 ± 0.12 ^e^	70.53 ± 0.15 ^d^	79.63 ± 0.76 ^ab^	6.73 ± 0.45 ^ab^	19.21 ± 0.32 ^b^
	5%ST	12.98 ± 0.99 ^a^	65.70 ± 0.10 ^b^	70.97 ± 0.06 ^c^	79.67 ± 0.47 ^ab^	6.50 ± 0.10 ^b^	18.18 ± 0.87 ^bc^
	7%ST	13.28 ± 0.48 ^a^	66.00 ± 0.10 ^a^	71.27 ± 0.15 ^b^	80.20 ± 0.36 ^a^	6.67 ± 0.06 ^a^	18.03 ± 0.31 ^c^
	10%ST	12.41 ± 0.41 ^b^	66.17 ± 0.23 ^a^	71.53 ± 0.06 ^a^	79.63 ± 0.15 ^b^	6.37 ± 0.12 ^b^	18.23 ± 0.54 ^c^
	3%XG	15.30 ± 1.58 ^a^	65.13 ± 0.15 ^de^	70.57 ± 0.12 ^d^	79.43 ± 0.55 ^a^	6.57 ± 0.12 ^ab^	19.80 ± 0.78 ^ab^
	5%XG	16.04 ± 0.24 ^a^	65.40 ± 0.17 ^cd^	70.83 ± 0.15 ^cd^	79.80 ± 0.26 ^ab^	6.73 ± 0.06 ^a^	20.10 ± 0.47 ^a^
	7%XG	14.61 ± 0.44 ^a^	65.47 ± 0.06 ^c^	70.97 ± 0.12 ^c^	79.93 ± 0.78 ^ab^	6.73 ± 0.25 ^ab^	19.26 ± 0.47 ^ab^
	10%XG	12.62 ± 1.70 ^a^	66.20 ± 0.26 ^a^	71.60 ± 0.20 ^ab^	79.97 ± 0.32 ^ab^	6.53 ± 0.15 ^ab^	18.05 ± 1.11 ^bc^

Note: SRN, Sharuan Nian; PD, polydextrose; Δ*H*, enthalpy of gelatinization; *T*_p_, peak temperature; *T*_o_, onset temperature; *T*_c_, conclusion temperature; CK, the sample without PD. All the data are expressed as mean ± SD, number of repetitions—*n* = 3. Different superscript letters indicate the significant differences (*p* < 0.05) within the column for each early indica rice variety.

**Table 8:** Effect of adding polydextrose on thermal property of early indica rice flours determined on day 21.

Rice Variety	PD Addition	Δ*H* (J/g)	*T*_o_ (°C)	*T*_p_ (°C)	*T*_c_ (°C)	Peak Width (°C)	Peak Height (0.01 mW/mg)
IP46	CK	7.80 ± 0.23 ^a^	41.93 ± 0.91 ^c^	55.47 ± 0.15 ^d^	66.93 ± 0.55 ^b^	13.30 ± 0.10 ^a^	8.15 ± 0.16 ^a^
	3%ST	7.37 ± 0.06 ^b^	42.70 ± 0.44 ^c^	55.73 ± 0.35 ^cd^	67.63 ± 0.42 ^ab^	12.40 ± 0.35 ^bcd^	8.29 ± 0.24 ^a^
	5%ST	6.57 ± 0.12 ^d^	43.93 ± 0.38 ^b^	56.07 ± 0.47 ^bcd^	66.73 ± 0.91 ^bc^	12.57 ± 0.12 ^b^	7.27 ± 0.17 ^c^
	7%ST	6.65 ± 0.08 ^d^	44.53 ± 0.46 ^b^	56.63 ± 0.40 ^ab^	67.43 ± 0.12 ^b^	12.37 ± 0.06 ^c^	7.50 ± 0.01 ^c^
	10%ST	6.44 ± 0.18 ^d^	43.93 ± 0.25 ^b^	56.30 ± 0.30 ^bc^	67.90 ± 0.60 ^ab^	12.50 ± 0.17 ^bc^	7.18 ± 0.26 ^c^
	3%XG	7.04 ± 0.24 ^c^	44.30 ± 0.36 ^b^	56.00 ± 0.53 ^bcd^	67.20 ± 0.00 ^c^	12.23 ± 0.06 ^d^	7.94 ± 0.17 ^a^
	5%XG	6.52 ± 0.24 ^d^	44.47 ± 0.29 ^b^	56.53 ± 0.42 ^ab^	67.70 ± 0.62 ^abc^	12.13 ± 0.12 ^d^	7.56 ± 0.15 ^bc^
	7%XG	6.56 ± 0.26 ^cd^	44.90 ± 0.92 ^ab^	56.97 ± 0.47 ^ab^	67.27 ± 0.15 ^bc^	12.00 ± 0.30 ^d^	7.61 ± 0.06 ^b^
	10%XG	6.12 ± 0.31 ^d^	46.57 ± 0.76 ^a^	57.07 ± 0.38 ^a^	68.27 ± 0.21 ^a^	11.50 ± 0.17 ^e^	7.36 ± 0.25 ^bc^
SRN	CK	5.25 ± 0.39 ^a^	44.47 ± 0.21 ^b^	55.80 ± 0.72 ^bcd^	64.03 ± 0.06 ^b^	10.60 ± 0.26 ^c^	6.67 ± 0.34 ^a^
	3%ST	4.96 ± 0.29 ^ab^	42.50 ± 1.14 ^c^	54.60 ± 0.61 ^d^	64.33 ± 0.61 ^ab^	12.07 ± 0.21 ^a^	5.74 ± 0.35 ^b^
	5%ST	4.39 ± 0.58 ^abcd^	43.03 ± 0.51 ^c^	55.23 ± 1.50 ^abc^	64.87 ± 0.55 ^a^	11.70 ± 0.10 ^b^	5.10 ± 0.73 ^b^
	7%ST	4.51 ± 0.26 ^bc^	43.00 ± 0.87 ^c^	55.37 ± 0.25 ^d^	63.77 ± 0.21 ^b^	10.97 ± 0.35 ^c^	5.60 ± 0.28 ^bc^
	10%ST	4.21 ± 0.58 ^bcd^	43.73 ± 0.68 ^bc^	55.77 ± 0.58 ^cd^	64.00 ± 0.40 ^ab^	10.83 ± 0.21 ^c^	5.30 ± 0.59 ^bc^
	3%XG	4.50 ± 0.45 ^abcd^	46.00 ± 0.52 ^a^	56.50 ± 0.98 ^abc^	64.07 ± 0.42 ^ab^	9.93 ± 0.12 ^d^	6.13 ± 0.53 ^ab^
	5%XG	4.19 ± 0.17 ^cd^	45.77 ± 0.12 ^a^	56.93 ± 0.29 ^a^	64.17 ± 0.31 ^ab^	9.80 ± 0.17 ^de^	5.74 ± 0.09 ^b^
	7%XG	3.90 ± 0.29 ^d^	46.73 ± 0.85 ^a^	57.07 ± 0.68 ^ab^	63.83 ± 0.31 ^b^	9.53 ± 0.21 ^e^	5.53 ± 0.52 ^bc^
	10%XG	3.88 ± 0.17 ^d^	45.67 ± 0.75 ^a^	56.90 ± 0.20 ^a^	64.00 ± 0.40 ^ab^	10.03 ± 0.06 ^d^	5.15 ± 0.22 ^c^

Note: SRN, Sharuan Nian; PD, polydextrose; Δ*H*, gelatinization enthalpy; *T*_p_, peak temperature; *T*_o_, onset temperature; *T*_c_, conclusion temperature; CK, the sample without PD. All the data are expressed as mean ± SD, number of repetitions—*n* = 3. Different superscript letters indicate the significant differences (*p* < 0.05) within the column for each early indica rice variety.

**Table 9:** Effect of adding polydextrose on the paste aging of early indica rice.

PD Addition	Aging (%)		*T*_c_ Reduction Percent (%)	
	IP46	SRN	IP46	SRN
CK	52.35 ± 5.94 ^ab^	35.18± 1.49 ^a^	30.29 ± 2.65 ^a^	19.62 ± 0.47 ^b^
3%ST	55.41 ± 8.15 ^ab^	34.45 ± 0.83 ^a^	26.92 ± 1.23 ^abc^	19.20 ± 1.46 ^abc^
5%ST	49.93 ± 0.83 ^a^	34.13 ± 6.61 ^ab^	27.82 ± 0.91 ^ab^	18.58 ± 0.27 ^c^
7%ST	47.68 ± 1.78 ^ab^	33.96 ± 0.95 ^a^	26.70 ± 0.28 ^bc^	20.48 ± 0.31 ^a^
10%ST	52.21 ± 6.63 ^ab^	33.99 ± 5.64 ^ab^	25.70 ± 0.95 ^c^	19.63 ± 0.35 ^b^
3%XG	45.63 ± 4.07 ^ab^	29.80 ± 5.80 ^a^	27.06 ± 0.20 ^b^	19.34 ± 1.01 ^abc^
5%XG	44.99 ± 2.96 ^b^	26.17 ± 1.39 ^b^	26.41 ± 0.80 ^bcd^	19.59 ± 0.20 ^b^
7%XG	44.82 ± 2.18 ^b^	26.72 ± 1.91 ^b^	26.78 ± 0.47 ^bc^	20.13 ± 1.15 ^ab^
10%XG	40.23 ± 7.77 ^b^	31.18 ± 5.14 ^ab^	25.66 ± 0.61 ^d^	19.96 ± 0.78 ^ab^

Note: SRN, Sharuan Nian; PD, polydextrose; aging is the percent of enthalpy of gelatinization on day 21 divided by that on day 0; *T*_c_, conclusion temperature; CK, the sample without PD. All the data are expressed as mean ± SD, number of repetitions—*n* = 3. Different superscript letters indicate the significant differences (*p* < 0.05) within the column.

**Table 10:** The GLM analysis of thermal properties of early indica rice flours at day 0.

Factors	Level	Δ*H* (J/g)	*T*_p_ (°C)	*T*_o_ (°C)	*T*_c_ (°C)	Peak Width (°C)	Peak Height (0.01 mW/mg)
Rice	IP46	14.34 ± 0.25 ^b^	80.08 ± 0.04 ^a^	70.64 ± 0.10 ^a^	92.89 ± 0.40 ^a^	9.83 ± 0.05 ^a^	15.68 ± 0.17 ^e^
variety	SRN	14.15 ± 0.25 ^b^	70.85 ± 0.04 ^h^	65.49 ± 0.10 ^e^	79.76 ± 0.40 ^d^	6.65 ± 0.05 ^d^	19.05 ± 0.17 ^a^
PD	ST	13.62 ± 0.25 ^cd^	75.52 ± 0.04 ^d^	68.14 ± 0.10 ^c^	86.36 ± 0.40 ^c^	8.24 ± 0.05 ^b^	16.94 ± 0.17 ^d^
type	XG	14.87 ± 0.25 ^a^	75.41 ± 0.04 ^e^	67.99 ± 0.10 ^cd^	86.30 ± 0.40 ^c^	8.24 ± 0.05 ^b^	17.79 ± 0.17 ^b^
PD	0	14.99 ± 0.39 ^a^	74.95 ± 0.07 ^g^	67.83 ± 0.16 ^d^	88.02 ± 0.63 ^b^	8.37 ± 0.08 ^b^	18.22 ± 0.26 ^b^
(%)	3	14.67 ± 0.39 ^a^	75.18 ± 0.07 ^f^	67.81 ± 0.16 ^d^	85.94 ± 0.63 ^c^	8.31 ± 0.08 ^b^	17.42 ± 0.26 ^c^
	5	14.17 ± 0.39 ^bc^	75.39 ± 0.07 ^e^	68.17 ± 0.16 ^bc^	85.98 ± 0.63 ^c^	8.25 ± 0.08 ^b^	17.17 ± 0.26 ^cd^
	7	14.13 ± 0.39 ^bc^	75.73 ± 0.07 ^c^	68.09 ± 0.16 ^cd^	86.00 ± 0.63 ^c^	8.25 ± 0.08 ^b^	17.08 ± 0.26 ^cd^
	10	13.26 ± 0.39 ^d^	76.08 ± 0.07 ^b^	68.43 ± 0.16 ^b^	85.71 ± 0.63 ^c^	8.01 ± 0.08 ^c^	16.93 ± 0.26 ^cd^

Note: SRN, Sharuan Nian; PD, polydextrose; Δ*H*, gelatinization enthalpy; *T*_p_, peak temperature; *T*_o_, onset temperature; *T*_c_, conclusion temperature. All the data are expressed as mean ± SD, number of repetitions—*n* = 3. Different superscript letters indicate the significant differences (*p* < 0.05) within the column.

**Table 11:** The GLM analysis of thermal properties of early indica rice flours at day 21.

Factors	Level	Δ*H* (J/g)	*T*_p_ (°C)	*T*_o_ (°C)	*T*_c_ (°C)	Peak Width (°C)	Peak Height (0.01 mW/mg)	Aging (%)
Rice	Ip46	6.89 ± 0.05 ^a^	56.22 ± 0.12 ^bc^	43.92 ± 0.20 ^d^	67.40 ± 0.09 ^a^	12.43 ± 0.09 ^a^	7.70 ± 0.06 ^a^	49.39 ± 0.78 ^a^
variety	SRN	4.51 ± 0.05 ^h^	55.99 ± 0.12 ^cd^	44.54 ± 0.20 ^bc^	64.11 ± 0.09 ^e^	10.61 ± 0.09 ^e^	5.76 ± 0.06 ^h^	31.49 ± 0.78 ^e^
PD	ST	5.82 ± 0.05 ^d^	55.69 ± 0.12 ^e^	43.38 ± 0.20 ^ef^	65.76 ± 0.09 ^c^	11.93 ± 0.09 ^b^	6.68 ± 0.06 ^de^	42.88 ± 0.78 ^b^
type	XG	5.58 ± 0.05 ^e^	56.52 ± 0.12 ^a^	45.08 ± 0.20 ^a^	65.75 ± 0.09 ^c^	11.11 ± 0.09 ^d^	6.79 ± 0.06 ^d^	37.99 ± 0.78 ^d^
PD	0	6.53 ± 0.09 ^b^	55.63 ± 0.19 ^e^	43.20 ± 0.31 ^f^	65.48 ± 0.15 ^d^	11.95 ± 0.14 ^b^	7.41 ± 0.10 ^b^	43.77 ± 1.23 ^b^
(%)	3	5.97 ± 0.09 ^c^	55.71 ± 0.19 ^de^	43.88 ± 0.31 ^de^	65.81 ± 0.15 ^bc^	11.66 ± 0.14 ^c^	7.03 ± 0.10 ^c^	41.32 ± 1.23 ^bc^
	5	5.42 ± 0.09 ^f^	56.19 ± 0.19 ^bc^	44.30 ± 0.31 ^cd^	65.87 ± 0.15 ^bc^	11.55 ± 0.14 ^c^	6.42 ± 0.10 ^f^	38.69 ± 1.23 ^d^
	7	5.41 ± 0.09 ^f^	56.51 ± 0.19 ^ab^	44.79 ± 0.31 ^abc^	65.58 ± 0.15 ^cd^	11.22 ± 0.14 ^d^	6.56 ± 0.10 ^ef^	38.29 ± 1.23 ^d^
	10	5.16 ± 0.09 ^g^	56.51 ± 0.19 ^ab^	44.98 ± 0.31 ^ab^	66.04 ± 0.15 ^b^	11.22 ± 0.14 ^d^	6.25 ± 0.10 ^g^	40.12 ± 1.23 ^d^

Note: SRN, Sharuan Nian; PD, polydextrose; Δ*H*, gelatinization enthalpy; *T*_p_, peak temperature; *T*_o_, onset temperature; *T*_c_, conclusion temperature. All the data are expressed as mean ± SD, number of repetitions—*n* = 3. Different superscript letters indicate the significant differences (*p* < 0.05) within the column.

**Table 12:** Effect of adding polydextrose on thermo-mechanical property of flour dough in early indica rice.

Rice Variety	PD Addition	DDT (min)	DST (min)	C1–Cs (Nm)	C3 (Nm)	C3/C4
IP46	CK	1.550 ± 0.080 ^a^	8.650 ± 0.350 ^a^	0.101 ± 0.004 ^d^	1.881 ± 0.053 ^a^	1.027 ± 0.005 ^b^
	3%ST	0.700 ± 0.030 ^c^	0.500 ± 0.100 ^c^	0.219 ± 0.013 ^a^	1.780 ± 0.010 ^b^	1.045 ± 0.005 ^a^
	7%ST	1.017 ± 0.035 ^b^	1.317 ± 0.585 ^b^	0.113 ± 0.014 ^d^	1.540 ± 0.002 ^c^	1.023 ± 0.004 ^b^
	3%XG	0.740 ± 0.140 ^c^	1.277 ± 0.025 ^b^	0.184 ± 0.004 ^b^	1.798 ± 0.109 ^b^	1.044 ± 0.008 ^a^
	7%XG	0.787 ± 0.035 ^c^	0.517 ± 0.085 ^c^	0.156 ± 0.014 ^c^	1.553 ± 0.021 ^c^	1.013 ± 0.002 ^c^
SRN	CK	1.300 ± 0.447 ^d^	1.533 ± 0.503 ^c^	0.587 ± 0.303 ^a^	1.790 ± 0.040 ^a^	1.323 ± 0.043 ^ab^
	3%ST	2.427 ± 0.245 ^c^	8.500 ± 0.100 ^b^	0.033 ± 0.004 ^b^	1.626 ± 0.004 ^c^	1.338 ± 0.008 ^a^
	7%ST	8.137 ± 0.015 ^a^	9.100 ± 0.200 ^a^	0.000 ± 0.001 ^c^	1.520 ± 0.001 ^d^	1.294 ± 0.002 ^b^
	3%XG	1.417 ± 0.265 ^d^	1.950 ± 0.350 ^c^	0.581 ± 0.084 ^a^	1.654 ± 0.014 ^b^	1.222 ± 0.011 ^c^
	7%XG	7.117 ± 0.885 ^b^	9.300 ± 0.100 ^a^	0.006 ± 0.006 ^c^	1.475 ± 0.004 ^e^	1.289 ± 0.007 ^b^
Rice variety	PD addition	C3–C4 (Nm)	C5–C4 (Nm)	α (−0.01 Nm/min)	β (0.01 Nm/min)	γ (−0.01 Nm/min)
IP46	CK	0.050 ± 0.010 ^b^	0.929 ± 0.051 ^a^	6.40 ± 0.60 ^a^	22.60 ± 1.00 ^b^	1.80 ± 0.00 ^b^
	3%ST	0.077 ± 0.009 ^a^	0.706 ± 0.004 ^c^	0.90 ± 0.10 ^b^	48.23 ± 5.86 ^a^	1.90 ± 0.70 ^b^
	7%ST	0.035 ± 0.007 ^b^	0.642 ± 0.013 ^d^	1.00 ± 0.20 ^b^	50.10 ± 3.30 ^a^	4.80 ± 0.40 ^a^
	3%XG	0.076 ± 0.019 ^a^	0.750 ± 0.013 ^b^	0.70 ± 0.50 ^b^	47.20 ± 13.40 ^a^	1.40 ± 1.00 ^b^
	7%XG	0.020 ± 0.003 ^c^	0.605 ± 0.031 ^d^	0.90 ± 0.10 ^b^	56.00 ± 0.20 ^a^	1.10 ± 0.70 ^b^
SRN	CK	0.436 ± 0.048 ^a^	0.747 ± 0.046 ^a^	11.00 ± 0.35 ^a^	44.27 ± 12.12 ^a^	6.20 ± 2.09 ^a^
	3%ST	0.411 ± 0.008 ^a^	0.630 ± 0.002 ^b^	4.80 ± 0.20 ^c^	46.50 ± 3.90 ^a^	6.20 ± 2.20 ^a^
	7%ST	0.345 ± 0.002 ^b^	0.543 ± 0.025 ^c^	2.37 ± 0.15 ^d^	28.67 ± 1.72 ^b^	4.70 ± 0.10 ^c^
	3%XG	0.301 ± 0.015 ^d^	0.794 ± 0.007 ^a^	8.40 ± 1.00 ^b^	28.11 ± 0.50 ^b^	3.30 ± 0.50 ^d^
	7%XG	0.330 ± 0.008 ^c^	0.542 ± 0.007 ^c^	2.60 ± 0.40 ^d^	26.20 ± 1.20 ^b^	6.30 ± 0.70 ^b^

Note: SRN, Sharuan Nian; PD, polydextrose; DDT, dough development time; DST, dough stability time; C1–Cs, protein weakness; C3, maximal gelatinization torque; C3/C4, amylase activity; C3–C4, starch breakdown; C5–C4, starch setback; α, heating rate; β, gelatinization rate; γ, enzymatic degradation rate; CK, the sample without PD. All the data are expressed as mean ± SD, number of repetitions—*n* = 3. Different superscript letters indicate the significant differences (*p* < 0.05) within the column for each early indica rice variety.

**Table 13:** The GLM analysis of thermo-mechanical properties of early indica rice dough.

Factors	Level	DDT (min)	DST (min)	C1–Cs (Nm)	C3 (Nm)	C3/C4
Rice	IP46	1.057 ± 0.392 ^d^	3.485 ± 0.891 ^b^	0.146 ± 0.049 ^cd^	1.739 ± 0.011 ^b^	1.030 ± 0.007 ^d^
variety	SRN	3.616 ± 0.392 ^a^	5.319 ± 0.891 ^a^	0.299 ± 0.049 ^a^	1.642 ± 0.011 ^d^	1.298 ± 0.007 ^a^
PD	ST	2.522 ± 0.392 ^b^	4.933 ± 0.891 ^ab^	0.175 ± 0.049 ^bcd^	1.689 ± 0.011 ^c^	1.175 ± 0.007 ^b^
type	XG	2.152 ± 0.392 ^bc^	3.871 ± 0.891 ^ab^	0.269 ± 0.049 ^ab^	1.692 ± 0.011 ^c^	1.153 ± 0.007 ^c^
PD	0	1.425 ± 0.481 ^cd^	5.092 ± 1.091 ^ab^	0.344 ± 0.060 ^a^	1.835 ± 0.013 ^a^	1.175 ± 0.009 ^b^
(%)	3	1.321 ± 0.481 ^cd^	3.057 ± 1.091 ^b^	0.254 ± 0.060 ^abc^	1.715 ± 0.013 ^bc^	1.163 ± 0.009 ^bc^
	7	4.264 ± 0.481 ^a^	5.058 ± 1.091 ^ab^	0.069 ± 0.060 ^d^	1.522 ± 0.013 ^e^	1.155 ± 0.009 ^c^
Factors	Level	C3–C4 (Nm)	C5–C4 (Nm)	α (−0.01 Nm/min)	β (0.1 Nm/min)	γ (−0.01 Nm/min)
Rice	IP46	0.051 ± 0.009 ^e^	0.761 ± 0.014 ^b^	2.7 ± 0.3 ^e^	4.11 ± 0.30 ^a^	2.1 ± 0.3 ^d^
variety	SRN	0.377 ± 0.009 ^a^	0.667 ± 0.014 ^e^	6.7 ± 0.3 ^b^	3.63 ± 0.30 ^ab^	5.5 ± 0.3 ^a^
PD	ST	0.226 ± 0.009 ^bc^	0.699 ± 0.014 ^d^	4.4 ± 0.3 ^cd^	4.01 ± 0.30 ^ab^	4.3 ± 0.3 ^b^
type	XG	0.202 ± 0.009 ^d^	0.728 ± 0.014 ^c^	5.0 ± 0.3 ^c^	3.74 ± 0.30 ^ab^	3.3 ± 0.3 ^c^
PD	0	0.243 ± 0.011 ^b^	0.838 ± 0.017 ^a^	8.7 ± 0.4 ^a^	3.34 ± 0.37 ^b^	4.0 ± 0.4 ^bc^
(%)	3	0.216 ± 0.011 ^cd^	0.721 ± 0.017 ^cd^	3.7 ± 0.4 ^d^	4.25 ± 0.37 ^a^	3.2 ± 0.4 ^c^
	7	0.182 ± 0.011 ^d^	0.583 ± 0.017 ^f^	1.7 ± 0.4 ^f^	4.02 ± 0.37 ^ab^	4.2 ± 0.4 ^b^

Note: SRN, Sharuan Nian; PD, polydextrose; DDT, dough development time; DST, dough stability time; C1–Cs, protein weakness; C3, maximal gelatinization torque; C3/C4, amylase activity; C3–C4, starch breakdown; C5–C4, starch setback; α, heating rate; β, gelatinization rate; γ, enzymatic degradation rate. All the data are expressed as mean ± SD, number of repetitions—*n* = 3. Different superscript letters indicate the significant differences (*p* < 0.05) within the column.

**Table 14:** Effect of adding polydextrose on the sensory evaluation of cooked early indica rice.

Rice Variety	PD Addition	Smell (%)	Appearance Structure (%)	Palatability (%)	Taste (%)	Cool Rice Texture (%)	Total Score (%)
IP46	CK	13.98 ± 0.11 ^a^	15.88 ± 0.22 ^b^	15.94 ± 0.26 ^b^	16.88 ± 0.22 ^b^	2.92 ± 0.11 ^a^	65.60 ± 0.84 ^b^
	3%ST	13.92 ± 0.13 ^a^	15.66 ± 0.42 ^b^	16.34 ± 0.42 ^ab^	17.36 ± 0.42 ^ab^	2.92 ± 0.11 ^a^	66.20 ± 0.45 ^b^
	7%ST	14.34 ± 0.42 ^a^	16.40 ± 0.42 ^ab^	16.66 ± 0.42 ^a^	17.64 ± 0.42 ^a^	2.96 ± 0.05 ^a^	68.01 ± 1.23 ^a^
	3%XG	13.92 ± 0.13 ^a^	16.46 ± 0.46 ^a^	16.42 ± 0.43 ^ab^	17.44 ± 0.44 ^ab^	2.96 ± 0.05 ^a^	67.20 ± 0.81 ^ab^
	7%XG	13.92 ± 0.11 ^a^	17.00 ± 0.35 ^a^	16.62 ± 0.65 ^ab^	17.74 ± 0.42 ^a^	2.90 ± 0.14 ^a^	68.18 ± 0.82 ^a^
SRN	CK	15.98 ± 0.11 ^a^	18.67 ± 0.41 ^a^	23.60 ± 0.42 ^a^	17.98 ± 0.11 ^b^	3.98 ± 0.11 ^a^	80.21 ± 0.55 ^b^
	3%ST	15.64 ± 0.42 ^a^	18.34 ± 0.42 ^a^	23.94 ± 0.26 ^a^	18.68 ± 0.43 ^a^	4.04 ± 0.15 ^a^	80.64 ± 0.64 ^b^
	7%ST	16.32 ± 0.41 ^a^	18.66 ± 0.42 ^a^	24.32 ± 0.50 ^a^	18.64 ± 0.42 ^a^	4.02 ± 0.18 ^a^	81.98 ± 0.32 ^a^
	3%XG	15.96 ± 0.22 ^a^	18.66 ± 0.42 ^a^	23.66 ± 0.42 ^a^	18.96 ± 0.22 ^a^	4.02 ± 0.11 ^a^	81.18 ± 0.90 ^ab^
	7%XG	16.30 ± 0.41 ^a^	18.88 ± 0.18 ^a^	24.40 ± 0.42 ^a^	18.90 ± 0.17 ^a^	3.98 ± 0.04 ^a^	82.60 ± 0.42 ^a^

Note: SRN, Sharuan Nian; PD, polydextrose; CK, the sample without PD. All the data are expressed as mean ± SD, number of repetitions―*n* = 8. Different superscript letters indicate the significant differences (*p* < 0.05) within the column for each early Indica rice variety.

**Table 15:** The GLM analysis of sensory evaluation of cooked early indica rice.

Factors	Level	Smell (%)	Appearance Structure (%)	Palatability (%)	Taste (%)	Cool Rice Texture (%)	Total Score (%)
Rice	IP46	14.01 ± 0.05 ^e^	16.21 ± 0.08 ^d^	16.32 ± 0.07 ^e^	17.32 ± 0.06 ^e^	2.93 ± 0.02 ^c^	66.79 ± 0.14 ^f^
variety	SRN	16.03 ± 0.05 ^a^	18.65 ± 0.08 ^a^	23.92 ± 0.07 ^a^	18.52 ± 0.06 ^a^	4.00 ± 0.02 ^a^	81.14 ± 0.14 ^a^
PD	ST	15.03 ± 0.05 ^c^	17.27 ± 0.08 ^c^	20.13 ± 0.07 ^c^	17.86 ± 0.06 ^d^	3.47 ± 0.02 ^b^	73.77 ± 0.14 ^d^
type	XG	15.01 ± 0.05 ^c^	17.59 ± 0.08 ^b^	20.11 ± 0.07 ^c^	17.98 ± 0.06 ^c^	3.46 ± 0.02 ^b^	74.16 ± 0.14 ^b^
PD	0	14.98 ± 0.06 ^cd^	17.27 ± 0.09 ^c^	19.77 ± 0.09 ^d^	17.43 ± 0.07 ^e^	3.45 ± 0.02 ^b^	72.90 ± 0.17 ^e^
(%)	3	14.86 ± 0.06 ^d^	17.28 ± 0.09 ^c^	20.09 ± 0.09 ^c^	18.11 ± 0.07 ^bc^	3.49 ± 0.02 ^b^	73.81 ± 0.17 ^d^
	7	15.22 ± 0.06 ^b^	17.74 ± 0.09 ^b^	20.50 ± 0.09 ^b^	18.23 ± 0.07 ^b^	3.47 ± 0.02 ^b^	75.19 ± 0.17 ^c^

Note: SRN, Sharuan Nian; PD, polydextrose. All the data are expressed as mean ± SD, number of repetitions—*n* = 8. Different superscript letters indicate the significant differences (*p* < 0.05) within the column.

**Table 16:** The properties of two varieties of early indica rice.

Rice Variety	Moisture Content (%)	Percent of Milled Rice (%)	Head Rice Percent (%)	Kernel Length/Width Ratio	Taste Value of Milled Rice (%)	Free Fatty Acids (%)	Amylose (%)	Protein (%)
IP46	11.12 ± 0.14 ^a^	67.38 ± 0.03 ^a^	62.77 ± 2.54 ^b^	2.40 ± 0.00	71.33 ± 0.58 ^b^	32.03 ± 0.73 ^a^	18.95 ± 0.58 ^a^	8.58 ± 0.07 ^a^
SRN	10.60 ± 0.22 ^b^	63.60 ± 0.40 ^b^	72.43 ± 2.03 ^a^	3.00 ± 0.00	79.33 ± 0.58 ^a^	30.17 ± 1.95 ^a^	12.02 ± 0.66 ^b^	7.24 ± 0.07 ^b^

Note: SRN, Sharuan Nian. Data are expressed as mean ± SD. Different superscript letters indicate the significant differences (*p* < 0.05) within the column.

## Data Availability

The original contributions presented in the study are included in the article, further inquiries can be directed to the corresponding author.

## References

[1] Ray D.K., Mueller N.D., West P.C., Foley J.A. (2013). Yield trends are insufficient to double global crop production by 2050. PLoS ONE.

[2] Huang G., Guo L., Zeng Y., Huang S., Zeng Y., Xie X. (2024). Changes in the grain yield and quality of early indica rice from 2000 to 2020 in southern China. Agronomy.

[3] Laborte A.G., de Bie K.C., Smaling E.M., Moya P.F., Boling A.A., Van Ittersum M.K. (2012). Rice yields and yield gaps in southeast Asia: Past trends and future outlook. Eur. J. Agron..

[4] Deng N., Grassini P., Yang H., Huang J., Cassman K.G., Peng S. (2019). Closing yield gaps for rice self-sufficiency in China. Nat. Commun..

[5] NBSC (2021). China Statistical Yearbook.

[6] Li X.J. (2022). Chief-Edition. Grain Equilibrium Moisture Theory and Practice.

[7] Kumar A., Lal M.K., Bagchi T.B., Sah R.P., Sharma S.G., Baig M.J., Nayak A.K. (2024). Glycemic Index of Rice: Role in Diabetics.

[8] Lal M.K., Singh B., Sharma S., Singh M.P., Kumar A. (2021). Glycemic index of starchy crops and factors affecting its digestibility: A review. Trends Food Sci. Technol..

[9] Kamil K.M., Rohana A.J., Mohamed W.M.I.W., Ishak W.R.W. (2023). Effect of incorporating dietary fiber sources in bakery products on glycemic index and starch digestibility response: A review. Nutrire.

[10] Schuchardt J.P., Wonik J., Bindirch U., Heinemann H.K., Khors H., Schneider I., Möller K., Hahn A. (2016). Glycemic index and microstructure analysis of a newly developed fiber enriched cookie. Food Funct..

[11] Bailey R.L., Gahche J.J., Lentino C.V., Dwyer J.T., Engel J.S., Thomas P.R., Betz J.M., Sempos C.T., Picciano M.F. (2011). Dietary supplement use in the United States, 2003–2006. J. Nutr..

[12] do Carmo M.M.R., Walker J.C.L., Novello D., Caselato V.M., Sgarbient V.C., Ouwehand A.C., Andreollo N.A., Hiane P.A., dos Santos E.F. (2016). Polydextrose: Physiological function and effects on health. Nutrients.

[13] Wong K.Y., Thoo Y.Y., Tan C.P., Siow L.F. (2022). Moisture absorption behavior and thermal properties of sucrose replacer mixture containing inulin or polydextrose. Appl. Food Res..

[14] Li Q.E., Schmidt S.J. (2011). Use of ramping and equilibrium water-vapor sorption methods to determine the critical relative humidity at which the glassy to rubbery transition occurs in polydextrose. J. Food Sci..

[15] Chang X.H., Zhang B., Hao M.Y., Zhang J.F., Yin Q., Wu F.Y., Xie X.H. (2018). Effect of polydextrose on the retrogradation of rice starch gel. Sci. Technol. Food Ind..

[16] Liu C., Li X.Y., Song H.D., Li X.J. (2024). Moisture sorption isotherms of polydextrose and its gelling efficiency in inhibiting the retrogradation of rice starch. Gels.

[17] Chen L., Ren F., Zhang Z.P., Tong Q.Y., Rashed M.M.A. (2015). Effect of pullulan on the short-term and long-term retrogradation of rice starch. Carbohydr. Polym..

[18] Liu C., Wang M.Y., Song H.D., Cao Z.Y., Jie Y., Li X.J., Ning G.Q. (2024). A study on the mechanism of polydextrose moisture absorption analyzed by hailwood-horrobin equilibrium moisture model. Sci. Technol. Cereals Oils Foods.

[19] Leelayuthsoontorn P., Thipayarat A. (2006). Textural and morphological changes of Jasmine rice under various elevated cooking conditions. Food Chem..

[20] Li H., Zhang H., Zhao C.B., Zhang S.X., Guo H.W., Liu J.S. (2018). Effect of cellulose and polydextrose on gelatinization and rheological properties of corn starch. Food Ind..

[21] Jing Y., Wang W.X., Yang L.Z., Liu Y.Q., Shi M.M. (2020). Effect of resistant starch and polydextrose on the quality of steamed bread. Sci. Technol. Food Ind..

[22] Schirmer M., Jekle M., Arendt E., Becker T. (2012). Physicochemical interactions of polydextrose for sucrose replacement in pound cake. Food Res. Int..

[23] Wang S.J., Li C.L., Copeland L., Niu Q., Wang S. (2015). Starch retrogradation: A comprehensive review. Compr. Rev. Food Sci. Food Saf..

[24] Chang Q., Zheng B.D., Zhang Y., Zeng H.L. (2021). A comprehensive review of the factors influencing the formation of retrograded starch. Int. J. Biol. Macromol..

[25] AOAC (1980). Official Methods of Analysis.

[26] Gao G.B., Cao Z.Y., Li X.J., Duan Y.S., Ma J.Y., Zhang Q. (2023). Changes in intergranular air properties and processing quality of paddy stored in a large cooled warehouse. Appl. Food Res..

[27] Tao L.S., Qin W., Wei Z., Li X.J., Zhang H.Q. (2022). Effect of small-scale storage on the cooking property and fatty acid profile. Appl. Food Res..

[28] (2008). Rice–Determination of Amylose Content.

[29] (2002). Milk and Milk Products–Determination of Nitrogen Content–Routine Method Using Combustion According to the Dumas Principle.

[30] (2011). Inspection of Grain and Oils—Determination of Fat Acidity Value of Grain and Oilseeds.

[31] Singh N., Kaur L., Singh S., Singh S.K. (2005). Physicochemical, cooking and textural properties of milled rice from different Indian rice cultivars. Food Chem..

[32] Zhou Z.K., Robards K., Helliwell S., Blanchard C. (2007). Effect of storage temperature on cooking behaviour of rice. Food Chem..

[33] (2008). Inspection of Grain and Oils—Method for Sensory Evaluation of Paddy or Rice Cooking and Eating Quality.

[34] (2010). Determination of Pasting Properties of Rice–Rapid Visco Analyzer Method.

[35] Rosell C.M., Collar C., Haros M. (2007). Assessment of hydrocolloid effects on the thermo-mechanical properties of wheat using the mixolab. Food Hydrocoll..

[36] SPSS Inc. (2006). SPSS for Windows.

